# The molecular basis of intrinsic resistance to azoles in *Rhizopus arrhizus*

**DOI:** 10.1128/aac.01337-25

**Published:** 2025-12-05

**Authors:** Michaela Lackner, Stephanie Toepfer, Mikhail V. Keniya, Carlos Lax, Francisco E. Nicolas, Victoriano Garre, Christoph Müller, Katharina Rosam, Lisa-Maria Zenz, Lucia Cesarini, Ulrike Binder, Joel D. A. Tyndall, Brian C. Monk

**Affiliations:** 1 Institute of Hygiene and Medical Microbiology, Medical University of Innsbruck 27280, Innsbruck, Austria; 2 Faculty of Dentistry, Sir John Walsh Research Institute, University of Otago 2495, Dunedin, Otago, New Zealand; 3 Departamento de Genética y Microbiología, Facultad de Biología, Universidad de Murcia 16751, Murcia, Spain; 4 Department of Pharmacy – Center for Drug Research, Ludwig-Maximilians Universität München 9183, Munich, Bavaria, Germany; 5 Department of Chemistry, Biology and Biotechnology, Università degli Studi di Perugia, Perugia, Umbria, Italy; 6 School of Pharmacy, University of Otago 2495, Dunedin, Otago, New Zealand; University of Iowa, Iowa City, Iowa, USA

**Keywords:** resistance mechanism, amino acid substitution, mucorales, antifungal resistance

## Abstract

The fungal disease mucormycosis, while generally regarded as rare and not transmitted between individuals, has become increasingly prevalent in disaster areas, among the immunocompromised, and in diabetics especially in response to COVID-19. Treatment options are limited. These include debridement of necrotizing tissue followed by complicated multicomponent therapies with amphotericin B and selected azole drugs, usually having poor outcomes. Mucormycetes are intrinsically resistant to the widely used short-tailed azole drugs fluconazole and voriconazole, but susceptible to the long-tailed, though expensive, azole posaconazole. Knowledge of the crystal structure of *Saccharomyces cerevisiae* sterol 14α-demethylase (Erg11, Cyp51) led to the hypothesis that this pattern of intrinsic azole resistance and susceptibility is due to the *Rhizopus arrhizus* CYP51-F5 isoform residues F129 and A291, while the CYP51-F1 isoform residues Y127 and V291 confer susceptibility to both short- and long-tailed azole drugs. The heterologous overexpression of individual recombinant *R. arrhizus* CYP51 isoforms in a *S. cerevisiae* host, with or without the cognate NADPH-cytochrome P450 reductase (RaCPR), and selective genetic modification of CYP51-F5 have tested this hypothesis. Complementary gene deletion experiments in *Rhizopus microsporus* confirm that the amino acid residues that align with *R. arrhizus* CYP51-F5 F129 and A291 determine the resistance or susceptibility pattern of *R. arrhizus* to short-, medium-, and long-tailed azoles.

## INTRODUCTION

Mucormycosis is caused by species in the ancient fungal order Mucorales. The most common causes of mucormycosis are species of *Rhizopus*, *Mucor*, and *Lichtheimia*, followed by *Rhizomucor*, *Cunninghamella*, *Apophysomyces*, and *Saksenaea* (1–8). These filamentous fungi are widely dispersed saprophytes that cause opportunistic infection in the immunocompromised (e.g., patients with *diabetes mellitus*, cancer, and organ transplants) (9, 10) and can affect the nasal passages, eyes, brain, lungs, and gastrointestinal tract. Subcutaneous infections occur due to traumatic injuries or burns. The use of glucocorticoids is an emerging risk factor in the treatment of diabetic patients with advanced COVID-19 (11). The few treatment options for mucormycosis usually have limited success, and mortality rates remain high (32–80%), depending on patient site of infection and underlying disease in the patient (6, 8, 12, 13). Treatment often involves disfiguring debridement of necrotic tissue and the use of liposomal amphotericin B as primary therapy (14). Intravenous isavuconazole or delayed-release posaconazole of moderate strength is strongly recommended as step-down or salvage therapy for patients unresponsive to amphotericin B (14). Echinocandins are not active against mucormycetes (15, 16). The short-tailed azole voriconazole has been used as the drug of choice to treat a wide range of pathogenic molds but is ineffective against the mucormycetes (17–19). The limited range of treatments currently available has been described as a burden for patients, particularly those in low- and middle-income settings.

**Fig 1:**
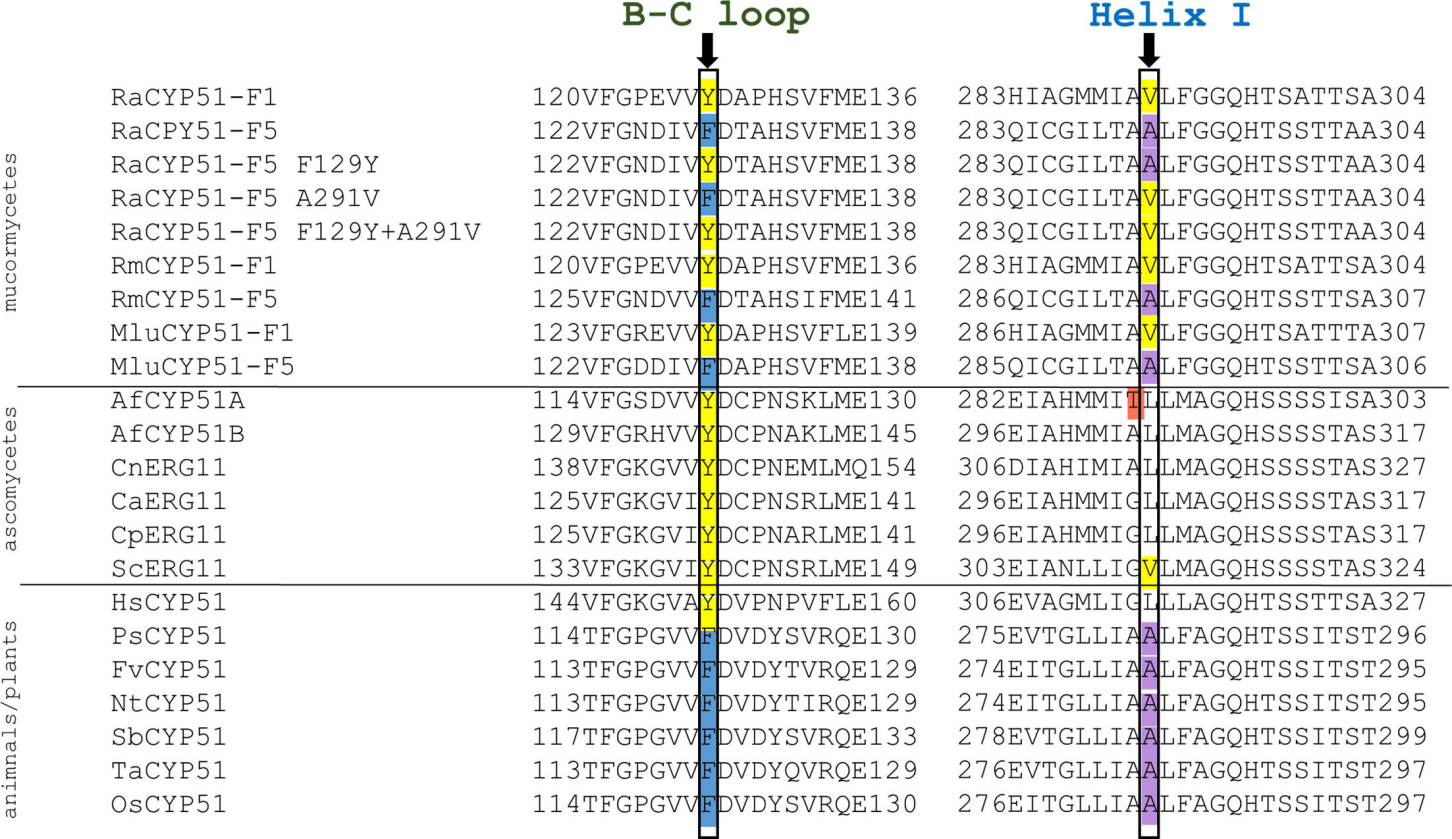
Alignment of B–C loop and helix I regions of CYP51 proteins. RaCYP51-F1 was aligned with RaCYP51-F5 and RaCYP51-F5 variants, as well as with CYP51s from *Rhizopus microsporus* (RmCYP51-F1 and RmCYP51-F5 isoforms), *Mucor lusitanicus* (MluCYP51-F1 and MluCYP51-F5), *Aspergillus fumigatus* (AfCYP51A and AfCYP51B), *Cryptococcus neoformans* (CnERG11), *Candida albicans* (CaERG11), *Candida parapsilosis* (CpERG11), *Saccharomyces cerevisiae* (ScERG11), *Homo sapiens* (HsCYP51), *Pisum sativum* (garden pea, PsCYP51), *Fragaria vesca* (wild strawberry FvCYP51), *Nicotiana tabacum* (tobacco NtCYP51), *Sorghum bicolor* (sorghum SbCYP51), *Triticum aestivum* (wheat TvCYP51), and *Oryza sativum* (rice OsCYP51), using CLC Sequence Viewer 8.0 software (Aarhus, Denmark). The arrows indicate the position of residues RaCYP51-F1 Y127 and V291 (highlighted in yellow), which are substituted in RaCYP51-F5 with F129 (highlighted in blue) and A291 (highlighted in magenta). The amino acid positions that correspond to RaCYP51-F5 F129 and A291 carry the amino acid variants phenylalanine and alanine in plant obtusifoliol 14α-demethylases. The residues equivalent to RaCPY51-F5 A291 are also substituted with the hydrophobic amino acid valine in RaCYP51-F1 and ScErg11 (highlighted in yellow) or leucine in pathogenic fungi. The helix I AfCYP51A T289 residue predominantly responsible for conferring innate fluconazole resistance on *A. fumigatus* is highlighted in red.

Acquired resistance due to azole exposure and agrochemicals has been widely studied. Molecular mechanisms responsible include drug target mutations, target overexpression, and drug efflux (20–28). In contrast, the intrinsic resistance of molds to fluconazole and other short-tailed azoles, despite their susceptibility to longer-tailed azoles (posaconazole), is poorly understood. Unlike yeast pathogens, which have a single CYP51 (often referred to as Erg11), most pathogenic molds have two or more sterol 14a-demethylase (CYP51) isoforms. In *Aspergillus fumigatus*, the functional expression of AfCYP51A and AfCYP51B (which share ~60% homology [29]) in *Saccharomyces cerevisiae*, along with the selective mutation of these isoforms, has demonstrated that innate resistance to fluconazole can be attributed predominantly to the helix I single amino acid substitution AfCYP51A T289 (30). This substitution borders the active site of CYP51, while an important instance of acquired resistance to short-tailed azoles can be linked to two amino acid changes in the CYP51A active site (i.e., Y121F in the BC loop and T289A in helix I, Fig. 1) (30). Such studies also show that voriconazole and longer-tailed azoles are bound by AfCYP51 at significantly higher affinities than fluconazole. In the present report, we experimentally expand and confirm the prediction from earlier homology modeling (Fig. 1, [(23]) that the F129 and A291 amino acid variants found in the mucormycete *Rhizopus arrhizus* CYP51-F5 (RaCYP51-F5) isoform confer intrinsic resistance to the short-tailed azole voriconazole. The prediction has been experimentally tested using physiological and biochemical analysis of recombinant full-length RaCYP51-F1 and RaCYP51-F5 isoforms (from the reference strain RA 99–880 [31]), functionally expressed in *S. cerevisiae*, and by mutating the two RaCYP51-F5 substitutions to the structurally aligned amino acids found in the voriconazole-susceptible RaCYP51-F1 (Fig. 1) isoform. Both isoform sequences are highly conserved among wild-type *R. arrhizus* strains (23, 32). Parallel gene deletion experiments in *R. microsporus* confirm this conclusion.

## RESULTS

### Homology modeling suggests how key substitutions in RaCYP51-F5 affect the binding and affinity of short- and long-tailed azole drugs

Homology models for RaCYP51-F1 and RaCYP51-F5 were prepared using the crystal structures of *S. cerevisiae* Erg11 in complex with the azole drugs fluconazole, voriconazole, and posaconazole (PDB IDs: 4WMZ, 5HS1, and 6E8Q, respectively) as templates and were compared. Interactions of individual RaCYP51 amino acids with fluconazole and voriconazole occur within the active site, while for posaconazole, they occur both within the active site and substrate entry channel (Table S1). Important interactions between these ligands, key water molecules, and the target are expected to occur at distances of ≤4 Å.

RaCYP51-F1 shows seven direct interactions with fluconazole with a closest approach ≤4 Å, plus two water-mediated hydrogen bonds. RaCYP51-F5 has seven comparable direct interactions with fluconazole, with the closest approach ≤4 Å, and one water-mediated hydrogen bond involving the main chain carbonyl N362 (equivalent to RaCYP51-F1 Q362 and ScErg11 S382). An additional close interaction involves RaCYP51-F5 M118, where this side chain adopts a more favorable interaction with fluconazole than in RaCYP51-F1 M116. Compared to RaCYP51-F1 Y127, the Y129F substitution in RaCYP51-F5 obviates a water-mediated hydrogen bond network with the fluconazole tertiary alcohol and a hydrogen bond with a heme propionate. This network is associated with resistance to short-tailed azoles in the ScErg11 F140Y mutant (33). Interaction with fluconazole may also be weakened by the helix I RaCYP51-F5 V291A substitution. The α-carbon of both residues is 3.9 Å from the 4-fluorine of the fluconazole difluorophenyl, but the V291 γ-carbon is 0.7 Å closer to the 4-fluorine than the A291 β-carbon (4.2 versus 4.9 Å). Fluconazole and voriconazole show comparable ≤4 Å interactions with RaCYP51-F1 and RaCYP51-F5, but voriconazole is 0.2–0.3 Å further from RaCYP51-F1 V291 and RaCYP51-F5 A291 than fluconazole. A water-mediated hydrogen bond network between fluconazole and RaCYP51-F1 Q362, as well as RaCYP51-F5 N362, is likely to involve the main chain carbonyls and the phenols of Y113 and Y115, respectively (Fig. S1A). Similarly, water-mediated hydrogen bond networks between voriconazole and RaCYP51-F1 F361 and Q362, as well as RaCYP51-F5 F361 and N362, are expected to involve their main chain carbonyls and amides, as well as the main chain carbonyls of M494 and M491, respectively (Fig. S1B). Posaconazole has six direct ≤4 Å interactions inside the active site in common with fluconazole and voriconazole, but has 10 additional closest approaches of ≤4.0 Å outside the active site in the substrate entry channel. This larger set of interactions is expected to compensate for the absence of water-mediated hydrogen-bond networks between posaconazole and the RaCYP51-F5 active site, i.e., via RaCYP51-F1 Y127; the main chains of the RaCYP51-F1 Q362 and RaCYP51-F5 N362 residues; the main chain carbonyls of RaCYP51-F1 M494 and RaCYP51-F5 M491; or the phenols of RaCYP51-F1 Y113 and RaCYP51-F5 Y115. In addition, the proximity of the posaconazole 4-fluoro group to the RaCYP51-F5 A291 substitution appears more comparable to fluconazole than voriconazole (Fig. S1C).

The models suggest that the relative affinity, and hence resistance, to the short-tailed azoles in *R. arrhizus* is dominated by interactions with the BC-loop RaCYP51-F5 Y129F substitution and affected to a lesser extent by interactions with the helix I RaCYP51-F5 V291A substitution (23, 32). Both effects could be neutralized by multiple additional interactions of the long tail of posaconazole with the substrate entry channel. We have tested these ideas by measuring susceptibility to azole ligands in functional recombinant isoforms of RaCYP51 and reverting key residues in the RaCYP51-F5 isoform to those found in RaCYP51-F1.

### Successful expression of recombinant RaCYP51-F1 and RaCYP51-F5 variants in *S. cerevisiae*

**Fig 2:**
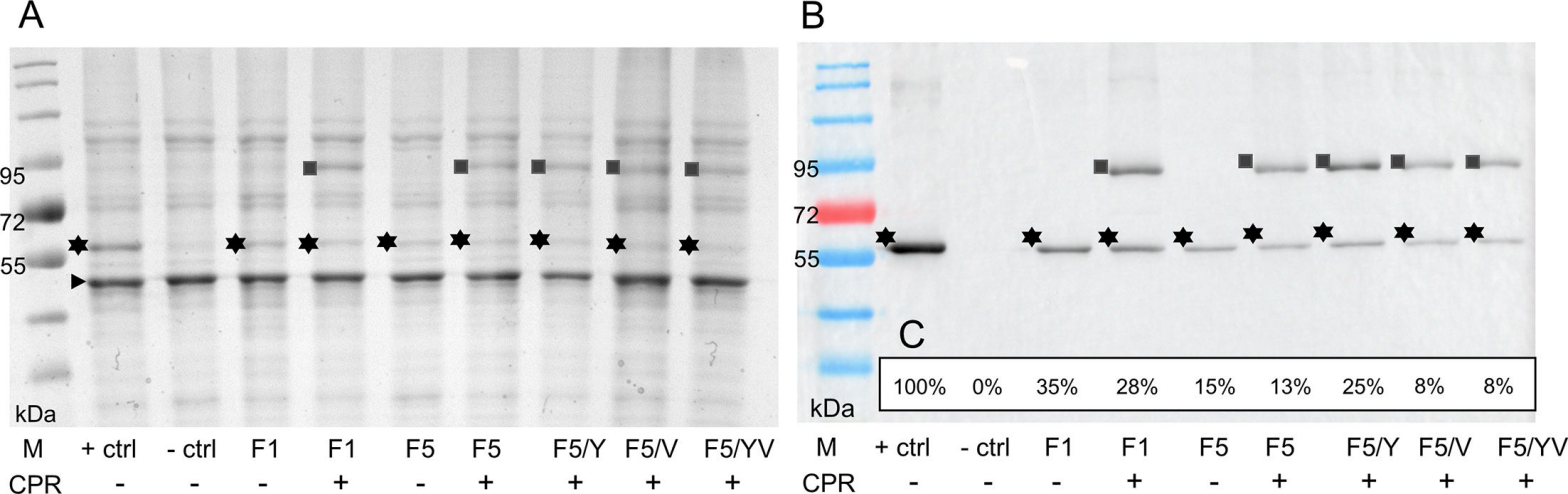
Protein expression profiles of recombinant *S. cerevisiae* variants overexpressing *R. arrhizus* CYP51-F1 or CYP51-F5 with or without cognate *R. arrhizus* NADPH-cytochrome P450 reductase CPR. (**A**) Coomassie stained SDS-PAGE and (**B**) corresponding Western blot obtained using anti-6×His antibody decoration with ECL detection. Fifteen micrograms of crude membrane preparation were loaded per lane. Preparations from the parental ADΔΔ derivative (Y1857) served as negative control (−ctrl) and strain Y2300 overexpressing ScErg11 as positive control (+ctrl). The following strains were used to characterize and quantify RaCYP51 expression: Y-F1(F1), Y-F1/CPR (F1 + CPR), Y-F5 (F5), Y-F5/CPR (F5 + CPR), Y-F5/Y/CPR (F5 F129Y + CPR), Y-F5/V/CPR (F5 A291V + CPR), and Y-F5/YV/CPR (F5 F129Y A291V + CPR). (**C**) Relative expression in % of the protein of interest on the Western blot. The expression of the positive control strain Y2300 was set to 100%. The square highlights the CPR protein (~82 kDa), the asterisk highlights the CYP51 protein (~60 kDa), and the triangle marks the most abundant, possibly tubulin-like, protein in crude membrane preparations that provided a loading control for normalization. For strain information, see Table S2. Lane M is the color prestained protein standard, broad range (10–250 kDa, New England Biolabs). The image was analyzed with ImageJ software (version 1.53k, Wayne Rasband and contributors, National Institutes of Health, USA, http://imagej.nih.gov/ij).

The expression of single genes in the heterologous expression system *S. cerevisiae* ADΔΔ allows for the analysis of individual gene effects without interference from background factors, such as efflux pump activity. This host is deleted of seven efflux pumps and is therefore hyper-susceptible to azole antifungals. This system has been successfully used to overexpress genes of interest from other yeasts (34–36), as well as molds such as *A. fumigatus* (30). The overexpression of *R. arrhizus* CYP51 in this host has not been reported previously; therefore, it was unclear whether it would be possible to clone *R. arrhizus* genes into the ADΔΔ yeast host, given their phylogenetic distance (37, 38). However, *Mucor lusitanicus* PDR transporters have been successfully transformed into this host (39), and Rosam et al. (40) have overexpressed CYP51 isoforms from the same species in the ADΔΔgal host strain (41). Major aims of the present study were to improve the system by overexpressing *R. arrhizus* CYP51 isoforms in the ADΔΔ host strain and by deleting the host *ScERG11*, rather than regulating its expression using the gal promoter (37, 38). Figure 2 shows the SDS-PAGE analysis with the corresponding Western blot of crude membranes prepared. Included were the host ADΔΔ strain; a control strain Y2300, which overexpresses His-tagged ScErg11; and strains expressing recombinant RaCYP51-F1 or RaCYP51-F5, with or without RaCPR. Coomassie staining detected protein bands expressed in the recombinant preparations at a molecular weight of ~60 kDa (asterisk, Fig. 2). An overexpressed protein band corresponding to the expected size of RaCPR was detected in all recombinant strains overexpressing CPR (82 kDa, square, Fig. 2). The expression of the recombinant constructs was confirmed by Western blotting using an anti-hexahistidine-tag-horse radish peroxidase conjugate to detect these proteins (Fig. 2B).

As expected, the crude membranes from the host strain did not produce recombinant protein, while strong expression was detected in the control strain Y2300, which was set at 100% expression for subsequent calculations. The recombinant strains expressing RaCYP51-F1 ± CPR showed the highest relative expression levels normalized to the tubulin-like protein used as a loading control (between 28% and 35%). All strains overexpressing RaCYP51-F5 had a lower relative expression signal than RaCYP51-F1 (Fig. 2B). Of these, RaCYP51-F5 F129Y + CPR had the highest relative expression level (25%), followed by RaCYP51-F5 (15%) and RaCYP51-F5 + CPR (13%). The lowest relative expression levels (Fig. 2B) were observed for RaCYP51-F5 A291V + CPR and the double mutant RaCYP51-F5 F129Y A291V + CPR (both 8%).

Tandem mass spectrometry (MS/MS) of tryptic fragments of Coomassie-stained protein bands confirmed the expected primary sequence of recombinant constructs in strains Y-F1/CPR and Y-F5/CPR (Fig. S2). High-level sequence coverage of between 66% and 82% was obtained for the constructs, including the GGR linkers and C-terminal hexahistidine tags in the case of the CPR, but not the CYP51 constructs. The fragment coverage confirmed Y127 in RaCYP51-F1 and the structurally aligned residue F129 in RaCYP51-F5, as well as V291 in RaCYP51-F1, but did not detect the structurally aligned residue A291 in RaCYP51-F5.

Generation times of recombinant strains (Table S3) were measured to assess strain fitness and the impact of genetic modifications. The generation times ranged from 2 to 2.7 h, comparable to that of the host strain (~2.2 h). Only strain Y-F1 showed a slightly lower generation time of ~3.5 h. These results suggest that the genetic modifications had no negative impact on the strain viability or fitness, and that the recombinant CYP51 proteins were successfully functionally expressed in the heterologous expression host ADΔΔ.

### Susceptibility testing indicates that RaCYP51-F5 plays a primary role in innate drug resistance

To evaluate the effects of azoles on the RaCYP51-F1 and RaCYP51-F5 homologs, as well as their variants, we conducted comparative susceptibility testing of the host strain Y1857 and the recombinant strains. While the term “short-, mid-, and long-tailed” azole is not a formally defined, it helps describe important structural properties of azoles that affect function: short-tailed azoles (fluconazole, voriconazole), mid-tailed azoles (isavuconazole), and long-tailed azoles (posaconazole, itraconazole) were categorized by the length of the tail anchoring them in the substrate entry channel.

**Fig 3:**
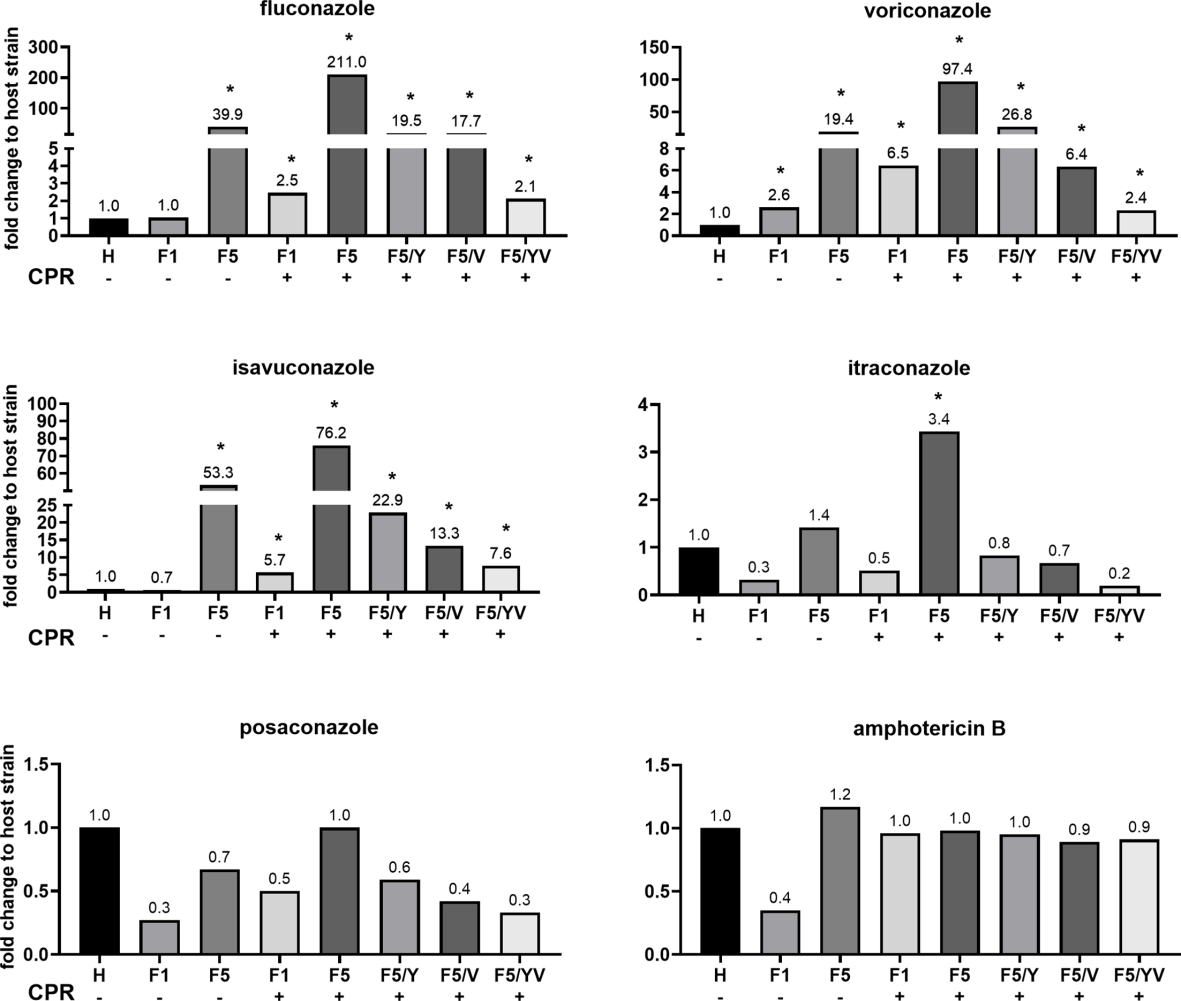
Fold change susceptibility of recombinant *S. cerevisiae* strains expressing RaCYP51 variants ± CPR compared to ADΔΔ. MIC_80_ values were obtained in SD medium at pH 6.8, and fold change was calculated in comparison to the host strain. Asterisk (*) above bars denote ≥2 fold changes, which are considered significant. Variations within a 2-fold range (± 1 doubling dilution) are generally accepted as technical variability and therefore not significant (42). The MIC_80_ ± standard deviation for ADΔΔ in [µM] was: fluconazole, 2.1 ± 0.2; voriconazole, 0.05 ± 0.0; isavuconazole, 0.05 ± 0.02; itraconazole, 0.06 ± 0.0; posaconazole, 0.1 ± 0.03; and amphotericin B, 1.8 ± 0.2. The MIC data of biological triplicates are shown in Table S4. For detailed strain information, see Table S2. Abbreviations: host strain (H), F5 F129Y (F5/Y), F5 A291V (F5/V), and F5 F129Y A291V (F5/YV).

In comparison to the host strain Y1857 (H in Fig. 3), the expression of RaCYP51-F5 gave a stronger resistance profile than expression of RaCYP51-F1, except for amphotericin B. As expected from previous studies, this effect was enhanced by the co-expression of RaCPR. The strain overexpressing RaCYP51-F1 + CPR was more resistant (up to two dilution steps) to azoles than RaCYP51-F1, and RaCYP51-F5 + CPR showed even higher relative increases (up to 5-fold) in MICs compared to RaCYP51-F5. The single amino acid substitutions F129Y and A291V gave resistance profiles similar to the strain expressing RaCYP51-F5. However, mutant strain Y-F5/Y/CPR tended to exhibit higher MICs than Y-F5/V/CPR, with the most pronounced difference observed for voriconazole, where Y-F5/Y/CPR showed a 4-fold increase in MIC compared to Y-F5/V/CPR. For the other azoles tested, the difference did not exceed a 1.7-fold change and was therefore not considered significant.

The strain carrying the double mutation F129Y A291V, which mimics wild-type RaCYP51-F1, exhibited similar susceptibilities as RaCYP51-F1. One exception was isavuconazole, where the susceptibility pattern for RaCYP51-F5 F129Y A291V + CPR (double mutant) did not match RaCYP51-F1 but was similar to RaCYP51-F1 + CPR. All strains showed MICs for the control antifungal, amphotericin B, comparable to host strain Y1857, which expresses ScERG11.

To better understand the molecular basis of innate resistance and susceptibility to azole drugs, the MIC_80_ ratio of the recombinant strains expressing RaCYP51-F5 + CPR and their variants was analyzed against RaCYP51-F1 + CPR (Y-F1/CPR) (Table 1). The expression of RaCYP51-F5 + CPR in strain Y-F5/CPR conferred 85.6-fold greater resistance to fluconazole than RaCYP51-F1 + CPR. For voriconazole, resistance was increased 15-fold. The triazole isavuconazole gave 13.3-fold increases in resistance, itraconazole 6.7-fold, and posaconazole 2.0-fold. The control antifungal, amphotericin B, gave <2-fold increases in resistance. These results clearly imply that RaCYP51-F5, in the presence of its cognate reductase, confers resistance to triazole drugs, an effect which is most prominent in short-tailed triazoles. The data in Table 1 also demonstrates that the RaCYP51-F5 + CPR F129Y, A291V, and F129Y + A291V mutations give substantial or complete complementation of the RaCYP51-F5 + CPR resistance phenotype.

**TABLE 1:** Fold change MIC_80_ of recombinant strains overexpressing RaCYP51-F5 variants + CPR compared to RaCYP51-F1 + CPR^*a*^

	Fold change MIC_80_ relative to RaCYP51-F1					
Variant	FLC	VRC	ISA	ITC	POS	AMB
RaCYP51-F5 Wild-type F129 A291	**85.6**	**15.1**	**13.3**	**6.7**	**2.0**	1.0
RaCYP51-F5 F129Y	**7.9**	**4.1**	**4.0**	1.6	1.2	1.0
RaCYP51-F5 A291V	**7.2**	1.0	**2.3**	1.3	0.8	0.9
RaCYP51-F5 F129Y A291V	0.9	0.4	1.3	0.4	0.7	0.9

^*a*^ Strains overexpressing RaCYP51 with: F1+CPR (Y-F1/CPR), F5+CPR (Y-F5/CPR), F5 F129Y+CPR (Y-F5/Y/CPR), F5 A291V+CPR (Y-F5/V/CPR), F5 F129Y A291V+CPR (Y-F5/YV/CPR). Fold change ≥2 (in bold) was considered as significant changes. Abbreviations: fluconazole (FLC), voriconazole (VRC), isavuconazole (ISA), itraconazole (ITC), posaconazole (POS), amphotericin B (AMB).

To support our findings and show the impact of CYP51-F5 in a wider context, we have also tested *Rhizopus microsporus* strains deleted of *CYP51-F1* and/or *CYP51-F5* for their azole susceptibility. The separate functional disruption of the *CYP51-F1* and *CYP51-F5* genes in *R. microsporus* confirmed that each gene can support the viability of this mucormycete but responds differentially to the presence of azole drugs, suggesting they have similar properties in *R. arrhizus*. The azole susceptibilities (Table 2) of the strains with *RmCYP51-F1* or *RmCYP51-F5* gene disruptions clearly demonstrate that the innate resistance of *R. microsporus* to voriconazole is associated with the *RmCYP51-F5* gene, not the *RmCYP51-F1* gene. The deletion of the *RmCYP51-F1* gene also conferred stronger resistance to isavuconazole, itraconazole, and posaconazole. In addition to the mutants above, a total of 34 clinical isolates of *Rhizopus* strains were profiled for their azole resistance. The median MIC_50_ values of all strains obtained were ~6 mg/L and >64 mg/L, for voriconazole and fluconazole, respectively; ~0.75 mg/L for isavuconazole; and ~1 mg/L for both itraconazole and posaconazole, confirming their resistance to the short-tailed azoles (Table 2). Currently, there are no clinical breakpoints established for mucormycetes. In contrast, clinical isolates of *A. fumigatus* are considered resistant if their MICs (mg/L) exceed the following breakpoints: 1 (voriconazole), 2 (isavuconazole), 1 (itraconazole), and 0.25 (posaconazole) (42).

**TABLE 2:** *In vitro* susceptibility of *R. microsporus* CYP51 deletion strains and clinical isolates of *Rhizopus* species^*a*^

		Median MIC (mg/L)				
		FLC	VRC	ISA	ITC	POS
Recombinant strains	RmΔCYP51-F5	> 16.0 ± 0.0	0.4 ± 0.12	0.25* ± 0.11	0.125* ± 0.05	0.25* ± 0.06
	RmΔCYP51-F1	> 16.0 ± 0.0	**1.0 ± 0.24**	2.0* ± 0.7	1.5* ± 0.5	0.5* ± 0.20
Clinical isolates	ATCC 11559	> 16.0 ± 0.0	8.0 ± 3.77	1.0 ± 0.24	2.0 ± 0.0	0.5 ± 0.24
	*R. arrhizus* (*n* = 19)	> 64.0 ± 0.0	8.0 ± 7.73	1.0 ± 1.54	2.0 ± 1.38	1.0 ± 0.55
	*R. microsporus* (*n* = 15)	> 64.0 ± 0.0	4.0 ± 4.75	0.5 ± 0.52	0.5 ± 1.97	0.5 ± 0.32

^*a*^ MIC_50_ values were measured after 24 h in biological triplicates ± standard deviation. For the CYP51 deletion strains, the median results of two strains tested per construct are shown. ATCC 11559 is the wild-type strain *R. microsporus*. For the clinical isolates, the median MIC_50_ of *R. arrhizus* (*n* = 19) or *R. microsporus* (*n* = 15) is shown.*MIC_90_ is shown instead of MIC_50_ because growth decreased from 100% to the MIC_90_ level. Two-way ANOVA comparing RmΔCYP51-F5 and RmΔCYP51-F1 identified significant (*P* value < 0.05) differences and is shown in bold. Statistical analysis was performed using GraphPad Prism version 10.2.3 for Windows, GraphPad Software, Boston, USA. Abbreviations: fluconazole (FLC), voriconazole (VRC), isavuconazole (ISA), itraconazole (ITC), posaconazole (POS).

### The *in vitro* assay using BOMCC as substrate shows enzyme-drug interactions of recombinant RaCYP51s

To determine the activity of recombinant RaCYP51s and the impact of different azoles on the protein, 7-benzyloxymethyloxy-3-cyanocoumarin (BOMCC) was used as an artificial CYP51 substrate. For this assay, strains must co-express their RaCPR; hence, strains Y-F1/CPR, Y-F5/CPR, Y-F5/Y/CPR, Y-F5/V/CPR, and Y-F5/YV/CPR were assayed (Table 3).

**TABLE 3:** Sensitivity of posaconazole-sensitive BOMCC hydrolysis by RaCYP51 variants to voriconazole and posaconazole^*a*^

	Posaconazole-sensitive BOMCC hydrolysis	
Strain variant	Posaconazole IC_50_ (µM)	Voriconazole IC_50_ (µM)
F1 + CPR	0.021 ± 0.011	0.100 ± 0.070
F5 + CPR	**0.056 ± 0.028**	**>1.0**
F5 F129Y + CPR	0.075 ± 0.035	0.140 ± 0.057
F5 A291V + CPR	**0.055 ± 0.007**	**0.500 ± 0.000**
F5 F129Y A291V + CPR	0.063 ± 0.000	0.060 ± 0.014

^*a*^ Strains overexpressing RaCYP51 plus RaCPR: Y-F1/CPR (F1+CPR), Y-F5/CPR (F5+CPR), Y-F5/Y/CPR (F5 F129Y+CPR), Y-F5/V/CPR (F5 A291V+CPR), Y-F5/YV/CPR (F5 F129Y A291V+CPR). The IC_50_ values are mean ± standard deviation obtained in two separate experiments. Two-way ANOVA comparing posaconazole IC_50_ and voriconazole IC_50_ identified significant differences (*P* value < 0.05) and is shown in bold. Statistical analysis was performed using GraphPad Prism version 10.2.3 for Windows, GraphPad Software, Boston, USA.

The assay detected an enzyme activity that generated a fluorescent hydroxycyanocoumarin product sensitive to 10 µM posaconazole. Each preparation yielded similar levels of enzyme activity that were also sensitive to voriconazole. Most importantly, the recombinant enzymes tested gave IC_50_ values at sub-µM concentrations that strongly mimicked the relative susceptibilities detected with whole cells (compare Table 3 with Fig. 3). The IC_50_ values indicated that the RaCYP51-F5 enzyme was ~2-fold less sensitive to posaconazole and >10-fold less sensitive to voriconazole than RaCYP51-F1. These differences for voriconazole were moderated in the RaCYP51-F5 F129Y enzyme, to a lesser extent in the RaCYP51-F5 A291V enzyme, and restored to the RaCYP51-F1 phenotype in the RaCYP51-F5 F129Y A291V enzyme.

### Affinity-purified RaCYP51-F5 protein exhibits type II binding of azole drugs

Type II binding studies that assess the affinity of azoles to proteins Cytochrome P450 proteins provide insight into how tightly such compounds fit into the fungal binding pocket and how mutations in the target protein affect binding compared to the wild type. A red shift of the Soret peak indicates coordination of the heme iron by the azole, distinguishing the azole-bound from the azole-free CYP51 (43). Our MIC data indicated that RaCYP51-F5 is responsible for azole resistance; therefore, we evaluated the binding of azoles to this enzyme.

**Fig 4:**
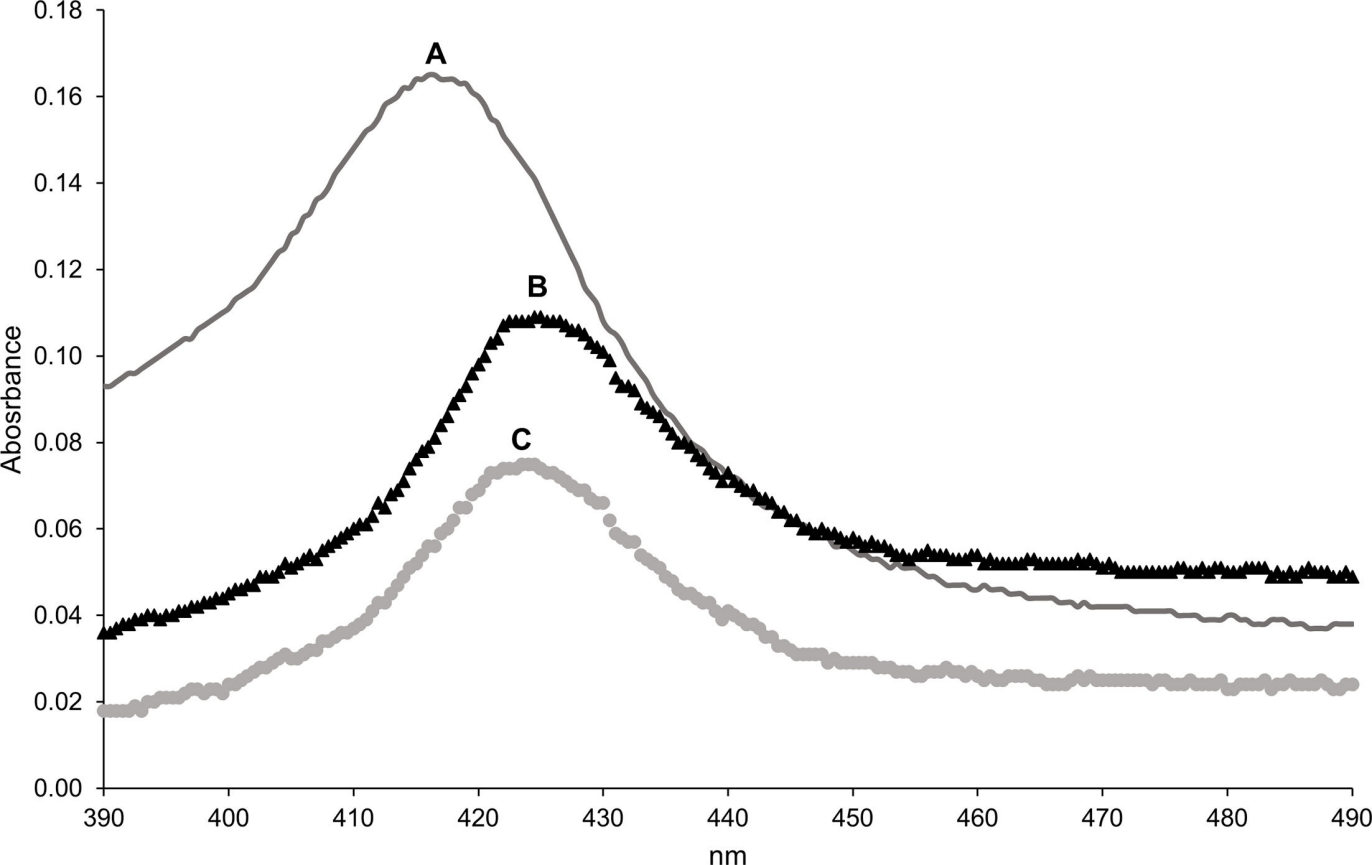
Spectrophotometric analysis of partially purified RaCYP51-F5. (**A**) Absolute spectrum of 1.0 µM RaCYP51-F5. (**B**) Type II difference spectrum in the presence of excess voriconazole. (**C**) Type II difference spectrum in the presence of excess posaconazole.

RaCYP51-F5, partially purified by Ni-NTA affinity chromatography, bound posaconazole or voriconazole upon exposure to saturating levels of these drugs. The absolute spectrum of 1.0 µM enzyme in the absence of azole ligands showed an absorbance peak at 418 nm, and difference spectra showed a red shift to 425 nm and 424 nm in the presence of 40 µM voriconazole or posaconazole, respectively (Fig. 4). This indicates, as expected, that the partially purified recombinant RaCYP51-F5 is capable of effective type II binding of both inhibitory ligands, despite being susceptible to posaconazole and showing strong resistance to voriconazole in *S. cerevisiae* cells expressing RaCYP51-F5.

### Azole inhibition of sterol biosynthesis confirms susceptibility patterns of recombinant strains

To assess antifungal drug efficacy and to phenotype RaCYP51 variants, sterol analysis with and without azole exposure (voriconazole and posaconazole) was performed.

The sterol analysis of the host strain Y1857 and recombinant strains detected 11 sterol intermediates (Table 4 and Table S5). The competence of recombinant enzymes in sterol biosynthesis and the effects of posaconazole and voriconazole exposure were analyzed by focusing on the relative production of ergosterol, lanosterol, and the toxic side product 14-methylergosta-8,24 (28)-dien-3,6-diol (MEDD). The accumulation of both lanosterol and MEDD during azole treatment of susceptible fungi was confirmed using the host strain Y1857, which expresses the native ScErg11. In untreated Y1857, ergosterol contributed 91% of total sterols, while lanosterol contributed 3% and MEDD was undetectable. When treated with 0.1 µM voriconazole or posaconazole, ergosterol content was reduced to 24% and 18%, whilst the lanosterol content increased to 38% and 36%. As expected, the toxic intermediate MEDD contributed 28% and 35% of total sterols.

**TABLE 4:** Effects of 0.1 µM voriconazole or posaconazole on sterol biosynthesis of strains carrying different CYP51 variants^*a*^

		Sterol content relative (%) to total sterols detected		
Strain	Treatment	Lanosterol	MEDD	Ergosterol
Y1857 parental strain	Voriconazole	38 ± 2	28 ± 1	24 ± 1
	Posaconazole	36 ± 1	35 ± 3	18 ± 2
	Control	3 ± 0	0 ± 0	91 ± 1
Y-F1 RaCYP51-F1	Voriconazole	33 ± 7	43 ± 1	14 ± 1
	Posaconazole	38 ± 8	47 ± 8	5 ± 0
	Control	35 ± 4	3 ± 7	21 ± 12
Y-F5 RaCYP51-F5	Voriconazole	25 ± 1	8 ± 2	62 ± 4
	Posaconazole	31 ± 3	50 ± 4	8 ± 1
	Control	16 ± 1	1 ± 1	79 ± 1
Y-F1/CPR RaCYP51-F1 + CPR	Voriconazole	22 ± 4	12 ± 3	62 ± 5
	Posaconazole	25 ± 2	59 ± 4	7 ± 2
	Control	18 ± 1	3 ± 1	75 ± 2
Y-F5/CPR RaCYP51-F5 + CPR	Voriconazole	9 ± 4	1 ± 1	86 ± 5
	Posaconazole	29 ± 2	48 ± 4	12 ± 3
	Control	4 ± 3	0 ± 0	89 ± 4
Y-F5/Y/CPR RaCYP51-F5 F129Y + CPR	Voriconazole	10 ± 1	1 ± 0	84 ± 1
	Posaconazole	27 ± 3	51 ± 4	11 ± 1
	Control	4 ± 1	0 ± 0	88 ± 1
Y-F5/V/CPR RaCYP51-F5 A291V + CPR	Voriconazole	19 ± 3	10 ± 3	67 ± 8
	Posaconazole	25 ± 2	55 ± 5	10 ± 2
	Control	12 ± 4	3 ± 2	81 ± 7
Y-F5/YV/CPR RaCYP51-F5 F129Y A291V + CPR	Voriconazole	26 ± 0	18 ± 1	51 ± 1
	Posaconazole	26 ± 2	56 ± 4	9 ± 2
	Control	21 ± 2	26 ± 5	47 ± 4

^*a*^ Percentage contributions of lanosterol, 14-methylergosta-8,24(28)-dien-3,6-diol (MEDD) and ergosterol to total sterols detected in the *S. cerevisiae* host strain expressing ScErg11, and derivative *S. cerevisiae* strains expressing recombinant RaCYP51s. Pre-grown cultures were treated with 0.1 µM of the respective antifungal until an OD_600nm_ = 2 was reached. Equivalent volumes of DMSO were added to controls. The results are presented as the average of three independent experiments, comprising six technical replicates in total with ± standard deviation expressed in %. More detailed information on sterols detected that contributed <6% of sterol content is given in Table S5. Statistical analysis performed is available in Supplementary Data.

In recombinant strains expressing RaCYP51-F1 or RaCYP51-F5, the sterol pattern was significantly different (Table 4). Strain Y-F1 gave a sterol composition very different from the control host strain, with a low level of ergosterol (21%), a high level (35%) of lanosterol, and a basal level of MEDD (3%). In contrast, strain Y-F5 had near-normal levels of ergosterol (79%), an intermediate level of lanosterol (16%), and barely detectable levels of MEDD (1%). These results indicate that, in the absence of the native ScErg11, the ergosterol biosynthetic pathway is significantly slowed in the *S. cerevisiae* host when the expression of the recombinant RaCYP51-F1 supports growth, and less so when RaCYP51-F5 is expressed. This was also observed in the growth curves, in which RaCYP51-F1 had the longest generation time (Table S3). Both recombinant strains expressing RaCyp51s mimicked the final effect of posaconazole (0.1 µM) on the control strain by reducing ergosterol content and increasing both lanosterol and MEDD content. Voriconazole (0.1 µM) treatment of strain Y-F1 similarly mimicked the disruption of sterol metabolism observed in host strain Y1857 expressing ScErg11. In contrast, the same treatment of strain Y-F5 resulted in a modest ergosterol content of 62%, accompanied by a comparatively smaller increase in lanosterol, from 16% to 25%, and in MEDD, from 1% to 8%, compared to the untreated setting. These results are consistent with the susceptibility of recombinant RaCYP51-F1 to voriconazole and the considerable resistance conferred by RaCYP51-F5 (19-fold increase compared to host strain).

The limited ergosterol biosynthesis due to incomplete complementation of ScErg11 by RaCYP51-F1 or RaCYP51-F5 was substantially corrected in strains Y-F1/CPR and Y-F5/CPR which co-express RaCPR. The ergosterol content of untreated strain Y-F1/CPR was at 75% and that of Y-F5/CPR was 89%. In addition, compared to strains Y-F1 and Y-F5, their contents of the precursor lanosterol were substantially lowered from 35% to 18% and 16% to 4%, respectively. This indicates the importance, for both recombinant CYP51 enzymes, of interaction with their cognate NADPH-cytochrome P450 reductase to most effectively metabolize lanosterol. The MIC values obtained for this set of recombinant strains with posaconazole and voriconazole were reflected in the responses of their ergosterol biosynthesis pathways to these inhibitors. On treatment with 0.1 µM posaconazole, both Y-F1/CPR and Y-F5/CPR showed similar disruption of ergosterol biosynthesis, with diminished production of ergosterol (7% and 12%), increased amounts of the precursor lanosterol (25% and 29%), and increased production of the toxic product MEDD (59% and 48%). Unlike the effect of posaconazole, exposure of strains Y-F1/CPR and Y-F5/CPR to 0.1 µM voriconazole differently affected ergosterol biosynthesis. While strain Y-F1/CPR, expressing fully functional RaCyp51-F1, exhibited reduced ergosterol (62%) and increased lanosterol (22%) and MEDD (12%) contents, strain Y-F5/CPR, expressing fully functional RaCyp51-F5, behaved comparably to the no-drug control. Specifically, ergosterol content remained high at 86%, whereas lanosterol (9%) and MEDD (1%) remained low.

The sterol profiles of untreated strains Y-F5/Y/CPR and Y-F5/V/CPR showed that the RaCYP51-F5 F129Y mutation in strain Y-F5/Y/CPR did not modify lanosterol or MEDD production compared to the parental strain Y-F5/CPR, while the RaCYP51-F5 A291V mutation caused a 3-fold increase in lanosterol (12%) and detectable amounts of MEDDs (3%). In contrast, the RaCYP51-F5 F129Y A291V double mutation reduced the efficiency of RaCYP51-F5 and increased lanosterol levels 5-fold, reaching 21%. The RaCYP51-F5 double mutation incurred the production of MEDDs (26%) and depletion of ergosterol (47%) to levels found only with CYP51 inhibitors. All three RaCYP51-F5/CPR variants showed sterol profiles in response to posaconazole (0.1 µM) comparable to those of their parental strain Y-F5/CPR. In response to voriconazole (0.1 µM), the sterol profile of strain Y-F5/Y/CPR was essentially unchanged from Y-F5/CPR, while the profiles of strains Y-F5/YV/CPR and Y-F5/V/CPR more closely matched those of strains Y-F1/CPR and Y-F5. These results are consistent with the voriconazole MIC_80_ values of 5.2, 1.4, 0.3, and 0.125 µM obtained for strains Y-F5/CPR, Y-F5/Y/CPR, Y-F5/V/CPR and Y-F5/YV/CPR, respectively.

## DISCUSSION

Coordinate functional expression of recombinant RaCYP51-F1 and RaCYP51-F5 isoforms from the *PDR5* locus, functionally enhanced with a cognate NADPH-cytochrome P450 reductase (RaCPR) expressed from the *PDR15* locus, has been achieved using the *S. cerevisiae* host strain ADΔΔ (34). This host lacks seven ABC transporters and *PDR3*, which makes it hyper-susceptible to azoles. Analysis of *in vitro* antifungal susceptibility in this system demonstrated that the RaCYP51-F5 + CPR conferred innate resistance to the short-tailed azoles fluconazole (85-fold) and voriconazole (15-fold) compared to RaCYP51-F1 + CPR.

Furthermore, innate resistance to the potent mid-length-tailed triazole isavuconazole was also observed (13-fold), while it was moderately weaker for the long-tailed triazole itraconazole (7-fold). In contrast, the RaCYP51-F5 + CPR isoform only conferred 2-fold greater resistance to posaconazole. The innate resistance to all these compounds was substantially ameliorated by either the F129Y or A291V mutation in RaCYP51-F5, which are structurally aligned to the Y127 and V291 amino acid residues in RaCYP51-F1.

The strain overexpressing RaCYP51-F5 F129Y (Y-F5/Y/CPR), with wild-type A291 in place, was overall slightly (between 0.6- and 1.7-fold) more resistant to azoles than RaCYP51-F5 A291V (Y-F5/V/CPR), which contains the wild-type F129. The only exception was voriconazole, for which RaCYP51-F5 F129Y had a 5-fold increase in resistance compared to RaCYP51-F5 A291V (MIC_80_ = ~1.5 µM vs ~0.3 µM). However, the presence of both F129 and A291, as in strain Y-F5/CPR, appeared to have the greatest impact on resistance. The combination led not only to voriconazole resistance (MIC = ~5 µM), but also to increased resistance to fluconazole (>200-fold), isavuconazole (76-fold), and a minor increase for itraconazole (3-fold), but not to posaconazole.

Our results are consistent with increased RaCYP51 function due to more efficient delivery of electrons to each recombinant RaCYP51 via their cognate CPR. The concentrations of posaconazole required for susceptibility are reasonably consistent with the relative levels of expression detected in Western blots for RaCYP51-F1 in Y-F1/CPR and RaCYP51-F5 in Y-F5/CPR compared to ScErg11 in Y2300.

Modeling suggests that the increased susceptibility due to the A291V mutation is likely caused by increased hydrophobic interaction with the 4-fluorine of the dihalophenyl head group of the azole drugs (with the exception of isavuconazole where the 5-fluorine is likely to be important), and reduced structural constraints in the active site between helix I and the BC-loop, e.g., due to reduced stiffness in helix I (Fig. S1). Conversely, the F129Y substitution might also increase structural constraints in the BC-loop, as the hydroxyl group of Y129 is likely hydrogen-bonded to the propionate group (C) of the heme.

Susceptibility testing in the homologous system *R. microsporus* ΔCYP51-F1 and ΔCYP51-F5 (Table 2) also confirmed that RmCYP51-F5 confers not only a 2.5-fold increase in resistance to the short-tailed azole voriconazole, but also to isavuconazole and itraconazole (the same observation as seen in the heterologous expression system). However, neither deletion mutant showed a response to fluconazole, with both remaining resistant. This lack of effect is most likely due to the presence of efflux pumps, for which fluconazole is a preferred substrate (44, 45). We previously demonstrated the importance of the ScErg11 Y140F mutation in conferring low-level resistance to fluconazole and voriconazole (33) and the *C. parapsilosis* Erg11 Y132F mutation in conferring even stronger resistance to these azole drugs, but not to posaconazole (35). It is therefore expected that the innate RaCYP51-F5 F129 mutation in strain Y-F5/V/CPR conferred resistance to fluconazole (~18-fold), voriconazole (~6-fold), and isavuconazole (~13-fold), compared to the host strain ADΔΔ. Similar to the present study, expression of *A. fumigatus* CYP51 isoforms in *S. cerevisiae* showed that the AfCYP51A amino acid substitutions F121 T289 (which corresponds to RaCYP51-F5 F129 A290, Fig. 1) confer resistance to fluconazole, voriconazole, and isavuconazole, but not to posaconazole (30). Furthermore, a wide range of cultivated plants are naturally resistant to azole agrochemicals and show comparable substitutions at sites in their CYP51s homologous to RaCYP51-F5 F129 and A291 (Fig. 1). These results strengthen our demonstration that the F129 and A291 substitutions in the RaCYP51-F5 isoform play a pivotal role in the innate azole resistance of *R. arrhizus*.

The hypothesis was further supported by sterol composition patterns. Treatment with posaconazole or voriconazole increased the amount of lanosterol but reduced that of ergosterol. Azoles and lanosterol are competitive substrates of CYP51, and an inhibition leads to an accumulation of lanosterol, which was also seen in wild-type *R. arrhizus* (46). In yeasts, the toxic accumulation of MEDD correlates with cell growth arrest and is indicative of azole inhibition (47). Treatment with posaconazole resulted in a stronger accumulation of MEDD compared to voriconazole. In some strains (e.g., Y-F5/CPR and Y-F5/Y/CPR), voriconazole exposure did not alter the sterol profile relative to untreated conditions. This implies that the amino acid A291 in wild-type RaCYP51-F5 contributes to resistance against short-tailed azole voriconazole more strongly than the Y129 mutation. We suggest that the A291V substitution affects lanosterol binding, probably due to adverse interaction of the RaCYP51-F5 V291 with the hydrophobic side chain of lanosterol. Competition between type I (lanosterol) and type II (azole) binding, measured in affinity-purified RaCYP51-F1 and RaCYP51-F5 preparations, may enable future exploration of this idea.

Our data, obtained using molecular genetic methods, whole cells, membrane preparations, and partially purified RaCyp51 preparations, collectively lead to the conclusion that the mucormycete residues that align with F129 and A291 in RaCYP51-F5, together with efflux pump activity (39), confer innate resistance to azole drugs, especially azoles most strongly reliant on interactions with the CYP51-F5 active site.

### Summary

The functional expression of RaCYP51-F1 and RaCYP51-F5 isoforms in *S. cerevisiae* provides powerful tools to investigate the physiology and biochemistry of the interactions of these drug targets with their substrates and inhibitory ligands. This expression strategy has provided better understanding of the function of *R. arrhizus* CYP51, including its ability to metabolize lanosterol, bind azole drugs, and use the artificial substrate BOMCC. It also has shown how their spectrum of innate resistance to azole drugs is affected by azole affinity for the heme and interactions with the F129 and A291 residues in the BC-loop and I helix within the RaCYP51-F5 isoform active site; i.e., resistance is maximal for short-tailed azoles and mitigated by interactions of long-tailed azoles, such as posaconazole, within the substrate channel. Our new insights not only provide a rational basis for salvage therapy of mucormycosis with posaconazole and raise questions about the use of isavuconazole but will also assist the structure- and function-directed design of more effective antifungal inhibitors that are so urgently needed.

## MATERIALS AND METHODS

### Homology modeling

Homology models for *R. arrhizus* CYP51-F1 and CYP51-F5 isoforms in complex with fluconazole, voriconazole, and posaconazole were generated using MODELLER v10.3 (48) (http://salilab.org/modeller/modeller.html), based on the crystal structures of ScErg11 in complex with these ligands (PDB IDs: 4WMZ, 5HS1 and 6E8Q, respectively) (33, 34, 41) and a sequence alignment generated from T-coffee (49, 50) (https://www.ebi.ac.uk/Tools/msa/tcoffee/). A total of 20 models for each structure were built using the Loop Model protocol in MODELLER. All models were generated with fluconazole, voriconazole, or posaconazole bound to the heme group in the active site. Water was not included. The models with the lowest molpdf scores were selected for further analysis. Visual analysis was carried out using PyMOL software (Schrödinger Inc., NY, USA).

### Culture media

#### Yeast

*Saccharomyces cerevisiae* strains were grown at 30°C on Synthetic Defined (SD) medium, which contains 0.79 g/L complete supplement mixture (Formedium Ltd., Hunstanton, UK), 2% (wt/vol) glucose, and 0.67% (wt/vol) yeast nitrogen base without amino acids (Formedium Ltd.). Solidified media was supplemented with 1.8% (wt/vol) agar (Oxoid Ltd., Hampshire, UK). Yeast transformants were selected at 30°C on solidified SD media containing 2% (wt/vol) glucose and either 0.77 g/L uracil drop-out (QBioGene, Irvine, CA, USA) or 0.77 g/L histidine drop-out (Formedium) complete supplement mixture. Larger-scale cultures of yeast strains were grown in YPD medium containing 1% (wt/vol) Bacto yeast extract (Formedium Ltd.), 2% (wt/vol) Bacto peptone (Formedium Ltd.), and 2% (wt/vol) glucose.

#### Rhizopus microsporus

The strains generated were derived from the *R. microsporus* wild-type strain ATCC 11559. Spores were collected using rich YPG media (51). Where specified, this medium was supplemented with uridine (200 mg/L). Transformants of the auxotrophic strain UM1 with the *pyrF* template used in CRISPR/Cas9 disruption experiments were grown in minimal media supplemented with casamino acids (MMC) (52). Electroporated protoplasts were resuspended in ice-cold YPG medium supplemented with 0.5 M sorbitol (YNGS) for 90 min, centrifuged at 800 rpm, and resuspended in YNB plus 0.5 M sorbitol (YNBS). Transformants were selected on solidified MMC media containing 0.5 M sorbitol. All strains were grown at 30°C.

### Codon optimization of homologous *R. arrhizus* genes for expression in the heterologous host *S. cerevisiae*

The full genome sequence of *Rhizopus oryzae* (*R. arrhizus*) strain RA 99–880 (alternative strain IDs: ATCC MYA-4621/FGSC 9543/NRRL 43880) is annotated in databases of Mucorales genomes (UniProt, Broad Institute) and NCBI (31). These sequences were compared using the basic local alignment search tool (BLAST). Two homologous protein variants of sterol 14α-demethylase encoded by the two gene variants (RaCYP51-F1, Sequence ID: EIE87079.1 sterol 14α-demethylase and RaCYP51-F5 Sequence ID: EIE91884.1, hypothetical protein RO3G_16595) have been described in previous studies (23, 53). Alleles most closely matched to the azole-sensitive and resistant forms of CYP51 in *Rhizopus azygosporus* and *M. lusitanicus* were assigned as F1 and F5, respectively (53). The multiple alignment scoring used to identify the *R. arrhizus* NADPH-cytochrome P450 reductase (Cpr) is summarized in Table S6. The hypothetical protein most closely matching (80.4%) *Mucor ambiguus* Cpr (54) was chosen as a cognate reductase. The ORFs of the genes, with codon usage optimized for the *S. cerevisiae* host, were designed by ATUM (Newark, CA, USA) using in-house algorithms. Plasmids containing the codon-optimized ORFs RaCPR1 (pJ207:344683; 7029 bp), RaCYP51-F5 (pJ201:344684; 4220 bp), or RaCYP51-F1 (pJ201:344685; 4229 bp) were synthesized by ATUM. Plasmids were maintained in *Escherichia coli* strain DH5α. Plasmid maps and plasmid sequences are given in Fig. S3 through S5, Table S7. A 25-nucleotide upstream sequence matching the 3′ end of the *PDR5* promoter and a 30-nucleotide downstream sequence encoding the GGR linker, hexahistidine tag (6×His), and stop codon were attached to open reading frames (ORFs) using the PCR.

### Construction of recombinant yeast strains

The *S. cerevisiae* ADΔΔ (Y1857) and ADΔΔgal (Y2494) hosts were used to create the recombinant strains used in this study (Table S2). The host ADΔΔ is a derivative of AD1-8u (Y663) with the *URA3* and *HIS1* ORFs deleted (33). In the ADΔΔgal strain, the promoter of native *ERG11* is replaced with *GAL1* promoter. This allows expression of the native ScErg11 in the presence of galactose, but not glucose (41). The purpose of this strategy was to evaluate host strain viability when using RaCYP51 in the absence of the native lanosterol 14α-demethylase. A susceptibility comparison of these two host strains (ADΔΔ and ADΔΔgal) overexpressing RaCYP51 is shown in Fig. S6. Because MICs were similar, the strains derived from ADΔΔ were used in this work. The ADΔΔgal-derived strains were retained as backups.

The host strains ADΔΔ and ADΔΔgal are deleted of seven pleiotropic drug resistance (PDR) ATP-binding cassette (ABC) transporters and the *PDR3* transcriptional regulator, while the *PDR1* transcriptional regulator bears the gain-of-function mutation *pdr1-3*, which drives constitutive expression from the *PDR5* promoter that contains pleiotropic drug resistance elements (PDREs).

Strains of *S. cerevisiae* that express recombinant RaCYP51-F1 or RaCYP51-F5 (Table S2) were constructed using transformation cassettes containing C-terminal hexahistidine (6×His)-tagged ORFs of either RaCYP51-F1 or RaCYP51-F5, bordered upstream by the *PDR*5 promoter and downstream by the *PGK* terminator (tPGK). The terminator is followed by the recyclable auxotrophic His marker LoxP-promoter_AgTEF1_-*ScHIS1*-terminator_AgTEF1_-loxP (LoxHis) obtained from the Euroscarf pUG6 plasmid (Euroscarf, Oberursel, Germany), together with a *PDR5*-specific downstream sequence at the 3′ end. The transformation cassette for 6×His-tagged NADPH-cytochrome P450 reductase *RaCPR* gene was designed to integrate at the *PDR15* locus using arms matching upstream and downstream sequences of *PDR15*. The RaCPR construct contains a *PGK* terminator and is controlled by the *PDR5* promoter positioned 5′ to the *RaCPR* ORF to ensure coordinate constitutive expression with the *PDR5* locus. *ScURA3* was used as a selective marker downstream of the *PGK* terminator as described by Lamping et al. (55).

DNA fragments contributing to each transformation cassette were amplified using the Phusion U Multiplex PCR Master Mix system (Thermo Fisher Scientific, Waltham, MA, USA) with primers designed to give at least a 24 nucleotide overlap between neighboring fragments. PCR products were separated using agarose gel electrophoresis, excised bands of interest extracted using the NucleoSpin purification kit (Macherey-Nagel, Düren, Germany), and their DNA content quantitated by spectrophotometry using an Implen NanoPhotometer NP80 (Thermo Scientific, Schwerte, Germany) at 260 nm and 280 nm. The transformation cassettes were amplified by fusion PCR using equimolar amounts of fragments and suitable outside primers.

Either RaCPR (ORF size: 2169 bp), RaCYP51-F1 (ORF size: 1585), or RaCYP51-F5 (ORF size: 1576 bp) bearing cassettes (cassette sizes: CPR 4993 bp, CYP51-F1 4646 bp, CYP51-F5 4637 bp) were integrated in the corresponding loci of the ADΔΔ and ADΔΔ gal host strains by homologous recombination using the Alkali Cation yeast transformation kit (MP Bio, Burlingame, CA, USA). The presence and location of the desired gene in His+ (for PDR5::RaCYP51) or Ura+ (for PDR15::RaCPR) transformants were initially confirmed by colony PCR with ExTaq polymerase (TakaRa, Shiga, Japan), using flanking primer pairs—a forward primer located upstream of the integration site and a reverse primer at the beginning of the ORF, 785 bp or 1061 bp apart for *PDR5* or *PDR15*, respectively (Table S8). Amplicon size was confirmed using agarose gel electrophoresis. Genomic DNA was extracted from at least three transformants of each variant using the Yeast DNA Extraction Kit (Thermo Scientific, Scientific, Waltham, MA, USA), and the full cassette PCR was amplified using Phusion U with primers positioned outside the area integrated. Several transformants with confirmed DNA sequences were phenotypically tested (growth rate, ability to grow on 2.0% glycerol, and susceptibility to azole drugs and amphotericin B) to exclude abnormally grown and petite strains.

Coordinate expression of RaCYP51 isoforms and RaCPR was achieved using *PDR15::RaCPR* strains as recipients of RaCYP51 cassettes. The LoxPHis marker was deleted from the *PDR5* locus using the induced recombination method of Güldener et al. (56). In brief, the His + strain was transformed with shuttle vector pSH69 (Euroscarf, Oberursel, Germany), which contains the hygromycin B resistance factor (hphMX) selection marker. Transformants were grown overnight in galactose-containing media (YPD + 2.0% Gal) to express the GAL1 promoter-controlled LoxP-specific Cre recombinase, which gave ~70–80% exclusion of the LoxPHis fragment. After dilution (1:30,000), 100 µL of samples were spread on YPD agar plates and incubated for 3 days, resulting in colonies with 40–70% loss of the pSH69 plasmid. Individual colonies were patched on –His dropout SD, YPD + hygromycin B, and YPD plates. Patches that grew on YPD but not –His or + hygromycin media were subcloned, genomic DNA (gDNA) obtained, and the recombinant region integrated at the *PDR5* locus was amplified as described above. The expected 1,591 nucleotide reduction in the size of the region due to LoxPHis excision was confirmed by agarose gel analysis and DNA sequence determination.

Two pairs of mutagenic primers were used to PCR-amplify DNA fragments encoding the amino acid modifications F129Y and A291V in RaCYP51-F5, replacing RaCYP51-F5 F129 and/or A291 with the corresponding amino acids found in the RaCYP51-F1 isoform. Each primer pair contained complementary forward and reverse sequences with the mutated codon in the center (Table S8). For the introduction of single mutations, two fragments were amplified using an upstream and a mutagenic reverse primer pair and a mutagenic forward and a downstream reverse primer pair, with the gDNA of a verified RaCYP51-F5–expressing strain used as the PCR template. To engineer the strain expressing RaCYP51-F5 F129Y A291V, an intermediate fragment of 535 bp was amplified using an upstream forward and a downstream reverse mutagenic primer pair. Gel-purified fragments (~1 µg each) were mixed in equimolar ratios and transformed into the appropriate host strain using the method described above. Deletion of endogenous CYP51 from RaCYP51-expressing strains, containing either the wild-type or *GAL1* promoter, was achieved by site-specific recombination with a linear disruption cassette (2,115 n) containing a *ScHIS1* selective marker flanked by regions upstream and downstream of the *ScERG11* gene, as described by Monk et al. (41) (Fig. S7). Strain transformation and selection of ΔwtScERG11 transformants were done as described above.

For the new strains produced in this study, all DNA transformation cassettes and genes inserted at the *S. cerevisiae PDR5*, *PDR15,* and *ERG11* loci were PCR-amplified and confirmed by DNA sequence analysis performed at the Genetic Analysis Services facility (University of Otago, Dunedin, New Zealand).

### Preparation of crude membranes and Western blot analysis of His-tagged recombinant protein

Yeast cells were grown in 1 L cultures in baffled 3 L Erlenmeyer flasks or in 15 mL cultures in 50 mL flasks for microscale experiments. The cultures were grown in YPD medium at 30°C to OD_600nm_ = 8 with shaking at 200 rpm. Harvested yeast cells were broken using a bead-beating protocol and crude membranes prepared by differential centrifugation (33). The protein concentrations of crude membrane fractions were estimated using the Lowry DC (detergent-compatible) Protein Assay kit (Bio-Rad, CA, USA), with bovine serum albumin (Thermo Fisher) used as standard.

Samples containing 15 µg of crude membrane protein, separated by SDS-PAGE in 8% acrylamide gels at pH 8.5 using the method of Laemmli et al. (57), were stained with Coomassie blue R250 (0.1% Coomassie Brilliant Blue [wt/vol], 20% methanol [vol/vol], 0.5% acetic acid [vol/vol]) or electrotransferred with the Trans-Blot Turbo Transfer System (Bio-Rad) to 0.45 µm nitrocellulose membranes (Bio-Rad) following a standard protocol. The membranes were blocked with 0.3% Tween 20 and 10% milk powder in phosphate-buffered saline (PBS), incubated for 90 min using 0.5 U/membrane of peroxidase-conjugated anti-6×His mouse monoclonal antibody (Merck, Vienna, Austria), and washed with blocking buffer. The Clarity Western ECL Substrate kit (Bio-Rad) was used according to manufacturer’s guidelines to detect immunodecorated bands. A Vilber Fusion Absolute (Vilber Bio Imaging, Eberhardzell, Germany) imaging system and ImageJ software (58) were used to record and analyze the data.

The equivalence of protein loading of Western blot membranes was confirmed using Ponceau S staining, i.e., 0.1% (wt/vol) Ponceau S in 5.0% (vol/vol) acetic acid for 1 min, followed by destaining with water. A highly abundant *S. cerevisiae* band, comparable in size to tubulin (MW = 50 kDa), was visualized by Coomassie staining to provide a loading control protein for relative quantification.

### BOMCC assay of RaCYP51 activity in crude membranes

The assay of BOMCC hydrolysis by crude membranes overexpressing RaCYP51 isoforms and their mutants plus RaCPR was performed essentially according to Riley et al. (59). Crude membranes were washed by centrifugation with 50 mM potassium phosphate buffer at pH 8, and their protein content was determined. The samples were then stored as concentrated samples at −80°C in 50 mM potassium phosphate buffer (pH 8). Double dilution series of voriconazole and posaconazole in 50 mM potassium phosphate buffer (pH 8), containing 5 mM MgCl_2_ and 0.5% DMSO, was prepared as 20 µL samples in triplicate in black, flat-bottomed microtiter plates. These samples were incubated for 10 min at room temperature with 50 µL crude membranes (200 µg). Ten microliters of regenerating solution, containing 0.3 units of glucose-6-phosphate dehydrogenase and 67 mM glucose-6-phosphate, were added to each well, and the plate was incubated for 15 min at 35°C. Twenty microliters containing 0.75 mM BOMCC and 1 mM NADP+ in 50 mM potassium phosphate buffer (pH 8) with 5 mM MgCl_2_ were added. The plate was incubated at 35°C for 30 min, and the production of the fluorescent product 3-cyano-7-hydroxycoumarin was monitored at an excitation wavelength of 410 ± 8 nm and an emission wavelength of 459 nm ± 8 nm using a CLARIOstar Plus plate reader (BMG Labtech, Alphatech Systems Ltd., Auckland, New Zealand). The reaction was linear for 30 min and stopped by adding 100 µL of 0.5 M Tris buffer, and the fluorescent product was measured after 30-minute incubation at room temperature to allow membranes to settle. The activity sensitive to 10 mM posaconazole was considered the total activity available.

### Enzyme purification

Recombinant enzymes RaCYP51-F1, RaCYP51-F5, and RaCPR were extracted from crude membranes using 17 mM n-decyl-β-D-maltoside (DM, 10 × critical micelle concentration [cmc]) (Anatrace, OH, USA) and ultracentrifugation, then purified by nickel-nitrilotriacetic acid (Ni-NTA) affinity and size-exclusion chromatography (SEC), essentially as described by Monk et al. (41).

### Identification of recombinant proteins by mass spectrometry

Protein bands separated by SDS-PAGE in 8% acrylamide gels were excised, subject to tryptic digestion, and the resultant protein fragments analyzed by MS/MS using an Orbitrap mass spectrometer at the University of Otago Center for Protein Research. Protein fragments were identified using the Mascot protein database (Matrix Science) and used to generate primary sequence coverage data (Fig. S2).

### Spectral scan of CYP51 with and without azole exposure

RaCYP51-F5 was obtained by Ni-NTA affinity chromatography from DM extracts of crude membrane preparations, as described by Sagatova et al. (34). Difference spectra were generated using the absolute absorbance spectra for Ni-NTA affinity-purified RaCYP51-F5 samples, obtained in the presence or absence of excess voriconazole or posaconazole, with an Ultraspec UV/Visible Spectrophotometer (Amersham Biosciences).

### Selection of disruptants in *R. microsporous CYP51-F1* and *CYP51-F5* genes

Stable disruption of *R. microsporus CYP51-F1* and *CYP51-F5* genes (Δ*RmCYP51-F1* and Δ*RmCYP51-F5*) was achieved using the methods described by Lax et al. (60). In brief, *in vitro* assembled ribonucleoprotein (RNP) complexes formed by Cas9 and guide RNA, together with 38 bp microhomology repair templates (Fig. S8), were electroporated into target cells. The templates for homologous recombination-mediated repair were generated by PCR amplification of 3.5 kb of the *RmpyrF* locus using primers that included short (38 bp) tails corresponding to sequences flanking the cleavage site (Table S9). The Cas9 enzyme was guided by gRNA1 and gRNA2 (Fig. S8) to *RmCYP51-F1* and *RmCYP51-F5*, respectively, in the uracil auxotroph recipient strain UM1 (60). In a first transformation experiment, five and seven transformants were obtained for *RmCYP51-F1* and *RmCYP51-F5*, respectively. Screening by PCR identified correct integrative disruption of *RmCYP51-F1* and *RmCYP51-F5* by *RmpyrF*. A single positive transformant was obtained for *RmCYP51-F1* and two for *RmCYP51-F5* (Fig. S8A and B). Initial transformants were expected to be heterokaryons as *R. microsporus* spores are multinucleate. After five vegetative cycles on selective MMC medium, the homokaryotic status of the three transformants was assessed by PCR. The absence of PCR amplification of the DNA fragment corresponding to the wild-type *RmCYP51-F1* and *RmCYP51-F5* loci, along with the amplification of the expected 3.5 kb larger DNA fragment, confirmed that all three transformants were homokaryons for the disruption (Fig. S8C and D). The three transformants were named RmCYP51-F1Δ1, RmCYP51-F5Δ1, and RmCYP51-F5Δ2. In a second transformation targeting *RmCYP51-F1*, screening of the four transformants was used to select the correctly integrated homokaryonic mutant RmCYP51-F1Δ2. *Rhizopus microsporus* strains used in this study are listed in Table S10.

### Susceptibility of yeast strains to antifungal drugs

#### Antifungals

The antifungal agents used in the present study were as follows: posaconazole (Schering-Plow, Kenilworth, NJ, USA), isavuconazole (Basilea, Basel, Switzerland), itraconazole (Sigma-Aldrich, Rowville, Australia), fluconazole, voriconazole, amphotericin B (Sigma-Aldrich, St. Louis, MO, USA), hygromycin B (Carl Roth GmbH, Karlsruhe, Germany), and 5-fluoroorotic acid (Merck, Darmstadt, Germany).

#### Yeast

Broth microdilution assays of drug susceptibility were performed according to EUCAST guidelines (61). As the *S. cerevisiae* strains used do not grow in standard RPMI medium at 37°C, the following modifications were used. The incubation time was extended to 48 h with shaking at 100 rpm, the temperature was lowered to 30°C, and RPMI was replaced with SD media containing 0.79 g/L complete supplement mixture (Formedium), buffered with 10 mM 2-(*N*-morpholino)ethanesulfonic acid (MES) and 20 mM HEPES, and adjusted with Tris to pH 6.8. Broth microdilution assays were performed in 96-well microtiter plates (Cellstar Cat. No. 655180, Greiner Bio-One, USA) and evaluated by spectrophotometry after 48 h (CLARIOstar Plus). All experiments were conducted in three independent biological replicates. Minimal inhibitory concentration (MIC_80_) values were determined for azoles, echinocandins, and polyenes as 80% growth reduction compared with a non-drug treated control. Mean MIC_80_ values and standard deviations were calculated using GraphPad.

#### Rhizopus microsporus

In total, 34 clinical isolates of *R. arrhizus* (*n* = 19) and *R. microsporus* (*n* = 15) were tested. Strains tested included wild-type strain *R. microsporus* ATCC 11559 plus the deletion mutants UM 10, UM 11, UM 12, and UM 13. Antifungal broth microdilution susceptibility testing was conducted according to the EUCAST protocol for mold testing version 9.3.2 (62) with the following modifications. As some strains were auxotrophic for uracil and leucine, instead of RPMI, all strains were cultured in yeast nitrogen base (YNB) agar supplemented with uracil (200 µg/mL) and leucine (20 µg/mL) at pH 4.5 for 5 days at 30°C. Spores were counted using a Neubauer chamber, and the inoculum was adjusted to a final concentration of 2.5 × 10^5^ spores/mL. MIC_50_ (50% growth inhibition) and MIC_90_ (90% or more growth inhibition) were determined after 24 h and 48 h by visual inspection using a 5× magnification mirror. CBS277.49 (*M. lusitanicus*) and ATCC 204304 (*Aspergillus flavus*) were used as reference strains. All experiments involved three independent biological replicates.

### Sterol composition

The parental strain ADΔΔ (Y1857) and recombinant strains with this background (strains Y-F1, Y-F5, Y-F1/CPR, Y-F5/CPR, Y-F5/YV/CPR, Y-F5/V/CPR, and Y-F5/Y/CPR) were characterized for their sterol composition, with and without exposure to voriconazole or posaconazole. For detailed strain information, see Table S2.

#### Growth conditions

Strains recovered from −80°C stock cultures on YPD agar at 30°C for 48 h were precultured at 30°C in SD medium (pH 6.8) on an orbital shaker (Infors HT, Ecotron, Bottmingen, Switzerland) at 200 rpm for 16 h. Samples from precultures were used to inoculate 50 mL of CSM complete medium to an OD_600nm_ = 0.25. The long-tailed azole posaconazole (0.1 µM), the short-tailed azole voriconazole (0.1 µM), or an equivalent volume of antifungal solvent DMSO (= control) was added to the cultures. The cultures were incubated on an orbital shaker at 200 rpm and 30°C until cultures reached OD_600nm_ = 2. This incubation period varied between 10 and 14 h depending on the strain and antifungal tested. Cells were harvested at room temperature by centrifugation at 2,500 × *g* for 10 min, freeze-dried, and the dry weight of the pellet determined.

Sterols were extracted using the procedure of Müller et al. (63), with the modification that 5 mg samples of lyophilized mycelium were homogenized using a bead mill (Mo Bio Laboratories, Vortex Genie 2, G-560E, Carlsbad, CA USA) at maximum speed (2,700 rpm) for 120 s.

#### Gas chromatography-mass spectrometry (GC-MS) analysis of sterol TMS ethers

The sterol composition of yeast samples was determined by GC-MS, according to Müller et al. (63). A Varian 3800 gas chromatograph was coupled with a Saturn 2200 ion trap (IT) from Varian (Darmstadt, Germany). The autosampler was a CombiPal from CTC Analytics (Zwingen, Switzerland), and the injector was a Varian 1177 with split/splitless option (Darmstadt, Germany). The instrument was equipped with a 30 m × 0.25 mm × 0.25 µm Agilent VF5ms capillary column and a 10 m EZGuard column (Waldbronn, Germany). The carrier gas was helium 5.0 (Air Liquide, Düsseldorf, Germany) at a constant flow rate of 1.4 mL/min. The inlet injector temperature was maintained at 250°C with an injection volume of 1 µL (split ratio 1:2). The GC oven started at 50°C (1.0 min hold) and was ramped up to 260°C (heating rate 50 °C/min), followed by a gradient of 4 °C/min up to 310°C (hold time 0.3 min). The transfer line temperature was 270°C. The IT/MS was switched on after 10 min (solvent delay) and scanned at a mass range from 100 to 600 *m/z* (EI, 70 eV).

The cell lyophilizate was ground and dispersed in aqueous 2 M sodium hydroxide solution to obtain a suspension of 5.0 mg/mL. The work-up procedure and the identification of sterol trimethylsilyl (TMS) ethers were performed as described by Müller et al. (63). The base peak of each sterol TMS ether was taken as a quantifier ion for calculating the peak areas: for internal standard (IS) cholestane *m/z* 217, ergosta-5,8,22-trien-3β-ol (lichesterol) *m/z* 363, ergosta-5,7,22-trien-3β-ol (ergosterol) *m/z* 363, ergosta-7,22-dien-3β-ol *m/z* 343, ergosta-5,8,22,24 (28)-tetraen-3β-ol *m/z* 466, 14-methylergosta-7,24 (28)-dien-3β-ol m/z *379,* 14-methylergosta-8,24 (28)-dien-3β-ol (14-methylfecosterol) *m/z* 379, ergosta-5,7-dien-3β-ol *m/z* 365, ergosta-7,24 (28)-dien-3β-ol (episterol) *m/z* 343, 4,4,14-trimethylcholesta-8,24 (28)-dien-3β-ol (lanosterol) *m/z* 393, and 14-methylergosta-8,24 (28)-dien-3β,6α-diol *m/z* 467 (“diol”). Unknown sterol (ergosta-?,?-diendi-3ß,?-ol) showed a base peak of *m/z* 363 and molecular peak of *m/z* 558, which indicated a sterol backbone with two hydroxyl groups and two double bonds. The amount of each sterol was expressed as a percentage of the total sterols. The results represent the mean of six parallel technical measurements from three independent biological replicates.

## Data Availability

All the plasmid and strains generated for this project are available upon request from the corresponding author. All the data sets generated during this study are included within the article and supplementary information.

## SUPPLEMENTAL MATERIAL

Supplemental material aac.01337-25-s0001.docx Fig. S1 to S8; Tables S1 to 10.

ASM does not own the copyrights to Supplemental Material that may be linked to, or accessed through, an article. The authors have granted ASM a non-exclusive, world-wide license to publish the Supplemental Material files. Please contact the corresponding author directly for reuse.
