# Supplementary material for: The molecular basis of intrinsic resistance to azoles in Rhizopus arrhizus
Source: Antimicrob Agents Chemother. 2025 Dec 5;70(1):e01337-25. doi: 10.1128/aac.01337-25 (PMC12777571; doi:10.1128/aac.01337-25)
Supplement: Supplemental material — Fig. S1 to S8; Tables S1 to 10. [file aac.01337-25-s0001.docx]

## Supplementary Material Tables

**Table S1.** Distance of closest approach (Å) between RaCYP51-F1 and F5 isoforms models, their azole ligands and with key water molecules.

| Fluconazole | | | | |  | Voriconazole | | |  | Posaconazole | | |
| --- | --- | --- | --- | --- | --- | --- | --- | --- | --- | --- | --- | --- |
| Sc  ERG11 | Ra  CYP51-F1 | | Ra  CYP51-F5 | |  | Sc  ERG11 | Ra  CYP51-F1 | Ra  CYP51-F5 |  | Sc  ERG11 | Ra  CYP51-F1 | Ra  CYP51-F5 |
|  |  | |  | |  |  |  |  |  | V66 | M53 | F55 |
|  |  | |  | |  |  |  |  |  | 6.6 | 4.8 | 4 |
|  |  | |  | |  |  |  |  |  | A69 | A56 | A57 |
|  |  | |  | |  |  |  |  |  | 2.9 | 3 | 3 |
|  |  | |  | |  |  |  |  |  | V70 | I57 | I58 |
|  |  | |  | |  |  |  |  |  | 3.9 | 2.9 | 3.6 |
|  |  | |  | |  |  |  |  |  | Y72 | F58 | F61 |
|  |  | |  | |  |  |  |  |  | 3.4 | 3.6 | 3.7 |
|  |  | |  | |  |  |  |  |  | G73 | G59 | G62 |
|  |  | |  | |  |  |  |  |  | 3.5 | 3.5 | 3.5 |
|  |  | |  | |  |  |  |  |  | M74 | I60 | K63 |
|  |  | |  | |  |  |  |  |  | 4.5 | 4.7 | 4.7 |
|  |  | |  | |  |  |  |  |  | L96 | V83 | L85 |
|  |  | |  | |  |  |  |  |  | 4.9 | 7.1 | 5 |
| Y126 | Y113 | | Y115 | |  | Y126 | Y113 | Y115 |  | Y126 | Y113 | Y115 |
| Water maediated H-bond with Y-OH | | | | |  |  |  |  |  |  |  |  |
| 2.7/3.9 | 2.4/4 | | 2.5/3.5 | |  | 3.2 | 3 | 3.5 |  | 3.4 | 3 | 2.9 |
| Distance Y-OH to water/closest FLC C | | | | |  | Distance Y side chain to nearest VRC F | | |  | Distance Y side chain to nearest POS C | | |
| L129 | M116 | | M118 | |  | L129 | M116 | M118 |  | L129 | M116 | M118 |
| 4.8 | 6.2 | | 3.8 | |  | 4.6 | 5.5 | 4.1 |  | 3.8 | 3.8 | 3.5 |
| T130 | T117 | | T119 | |  | T130 | T117 | T119 |  | T130 | T117 | T119 |
|  |  | |  | |  |  |  |  |  | Serine side chain rotated | | |
| 4.9 | 5.2 | | 4.9 | |  | 4.1 | 4.2 | 4.2 |  | 4.7 | 4.8 | 6.1 |
|  |  | |  | |  |  |  |  |  | F134 | F121 | F123 |
|  |  | |  | |  |  |  |  |  | 3.6 | 4 | 3.4 |
| I139 | V126 | | V128 | |  | I139 | V126 | V128 |  | I139 | V126 | V128 |
| 3.3 | 4.5 | | 4.3 | |  | 3.6 | 4.7 | 4.5 |  | 3.2 | 4.3 | 3.7 |
| Y140 | **Y127** | | **F129** | |  | **Y140** | **Y127** | **F129** |  | **Y140** | **Y127** | **F129** |
| Water mediated H-bond with Y-OH | | | | |  | Water mediated H-bond with Y-OH | | |  | No water mediated H-bond | | |
| 2.8/4.3 | 2.6/4.4 | | 4.8 | |  | 2.7/4.0 | 2.6/4.0 | 4.3 |  | 3.8 | 3.7 | 4.3 |
| Distance to water/FLC-OH | | | | |  | Distance to water/VRC-OH | | |  |  |  |  |
| L147 | | F134 | | F136 |  | L147 | F134 | F136 |  | L147 | F134 | F136 |
| 6.7 | 4.6 | | 5.4 | |  | 6.9 | 5 | 5.5 |  | 6.8 | 4.8 | 4.7 |
| F236 | F217 | | F218 | |  | F236 | F217 | F218 |  | F236 | F217 | F218 |
| 3.8 | 3.6 | | 3.4 | |  | 3.7 | 3.7 | 3.7 |  | 3.3 | 3.4 | 3.4 |
|  |  | |  | |  |  |  |  |  | P238 | P219 | P220 |
|  |  | |  | |  |  |  |  |  | 4 | 3.9 | 3.9 |
|  |  | |  | |  |  |  |  |  | F241 | F222 | F223 |
|  |  | |  | |  |  |  |  |  | 4.1 | 4.4 | 4.6 |
| G310 | A290 | | A290 | |  | G310 | A290 | A290 |  | G310 | A290 | A290 |
| 3.7 | 3.7 | | 3.7 | |  | 3.7 | 3.8 | 3.8 |  | 4.2 | 3.6 | 3.6 |
| V311 | V291 | | A291 | |  | V311 | V291 | A291 |  | V311 | V291 | A291 |
| 3.9/4.1 | 3.9/4.2 | | 3.9/4.9 | |  | 4.2/4.6 | 4.2/4.4 | 4.2/5.2 |  | 3.9/4.3 | 3.9/4.4 | 3.9/4.9 |
| Distance α-carbon/nearest side chain C to 4F | | | | |  | Distance α-carbon/nearest side chain C to 4F | | |  | Distance α-carbon/nearest side chain C to 4F | | |
| G314 | G294 | | G294 | |  | G314 | G294 | G294 |  | G314 | G294 | G294 |
| 3.1 | 3.1 | | 3.1 | |  | 3.8 | 3.8 | 3.8 |  | 3.2 | 3.2 | 3.2 |
| G315 | G295 | | G295 | |  | G315 | G295 | G295 |  | G315 | G295 | G295 |
| 3.9 | 3.9 | | 3.9 | |  | 4.0 | 4.1 | 4.1 |  | 3.9 | 4 | 4 |
| T318 | T298 | | T298 | |  | T318 | T298 | T298 |  | T318 | T298 | T298 |
| 3.8 | 3.5 | | 3.4 | |  | 3.7 | 3.3 | 3.6 |  | 3.5 | 3.6 | 3.6 |
| L380 | I360 | | I360 | |  | L380 | I360 | I360 |  | L380 | I360 | I360 |
| 3.6 | 3.2 | | 3.5 | |  | 3.8 | 2.9 | 2.8 |  | 3.5 | 3.6 | 4.3 |
|  |  | |  | |  |  |  |  |  | H381 | F361 | F361 |
|  |  | |  | |  |  |  |  |  | 3.5 | 6 | 6 |
| S382 | Q362 | | N362 | |  | S382 | Q362 | N362 |  | S382 | Q362 | Q362 |
| Main chain CO water mediated H-bond | | | | |  | Main chain NH water mediated H-bond | | |  |  |  |  |
| 2.6/4.6 | 2.6/4.7 | | 2.6/4.6 | |  | 2.9/5.5 | 3.1/4 | 3.1/4 |  | 3.5 | 5.4 | 3.7 |
| Distance to water/closest FLC N | | | | |  | Distance to water/closest VRC N | | |  |  |  |  |
| L383 | M363 | | M363 | |  | L383 | M363 | M363 |  | L383 | M363 | M363 |
| 4.2 | 5.5 | | 6 | |  | 3.7 | 5.3 | 5 |  | 4.7 | 5.4 | 5.4 |
|  |  | |  | |  |  |  |  |  | F384 | M364 | M364 |
|  |  | |  | |  |  |  |  |  | 3.8 | 3.3 | 3.5 |
| C470 | C455 | | C453 | |  | C470 | C455 | C453 |  | C470 | C455 | C453 |
| 4.5 | 4.5 | | 4.6 | |  | 4.6 | 4.6 | 4.6 |  | 4.5 | 4.5 | 4.5 |
|  |  | |  | |  |  |  |  |  | F506 | Y491 | Y488 |
|  |  | |  | |  |  |  |  |  | 4.2 | 4.2 | 4.2 |
|  |  | |  | |  |  |  |  |  | T507 | T492 | T489 |
|  |  | |  | |  |  |  |  |  | 3.8 | 2.9 | 3 |
|  |  | |  | |  |  |  |  |  | S508 | S493 | T490 |
|  |  | |  | |  |  |  |  |  | 3.3 | 3.3 | 3.3 |
| M509 | M494 | | M491 | |  | M509 | M494 | M491 |  | M509 | M494 | M491 |
|  |  | |  | |  | Main chain CO water mediated H bond | | |  |  |  |  |
| 3.3 | 4 | | 3.2 | |  | 3.0/4.2 | 3.1/4.0 | 3.1/4.1 |  | 3 | 3.2 | 3.2 |
|  |  | |  | |  | Distance to water/closest VRC N | | |  |  |  |  |
|  |  | |  | |  | V510 | V495 | V492 |  |  |  |  |
|  |  | |  | |  | 4.9 | 5.8 | 5.1 |  |  |  |  |

**Table S2.** Parental and recombinant *S. cerevisiae* strains used in this study.

| Strain | Strain abbreviation | Genotype | Reference |
| --- | --- | --- | --- |
| Y663  ADΔ |  | MATα *pdr1-3 Δyor1::hisG Δsnq2::hisG Δpdr3::hisG Δpdr10::hisG Δpdr11::hisG Δycf1::hisG Δpdr5::hisG Δpdr15::hisG Δpdr5*::*hisG Δpdr15*::*hisG* *Δura3::dpl200* | (1) |
| Y1857  ADΔΔ |  | ADΔ, *Δhis1*::*dpl200* | (2) |
| Y2411 |  | ADΔΔ, *Δpdr5::URA3* | (3) |
| Y2494 |  | ADΔΔ proERG11::proGAL1 | (3) |
| Y2300 |  | ADΔΔ PDR5::ScERG11 ΔScErg11::HIS1 | (2) |
| Y2645 | Y-F1 | ADΔΔ PDR5::RaCYP51-F1-6xHIS ΔScERG11::LoxP-HIS1 | This study |
| Y2647 | Y-F5 | ADΔΔ PDR5::RaCYP51-F5-6xHIS ΔScERG11::LoxP-HIS1 | This study |
| Y2691 |  | ADΔΔ PDR5::RaCYP51-F5-6xHIS F129Y ΔScERG11::LoxP-HIS1 | This study |
| Y2689 |  | ADΔΔ PDR5::RaCYP51-F5-6xHIS A291V ΔScERG11::LoxP-HIS1 | This study |
| Y2687 |  | ADΔΔ PDR5::RaCYP51-F5-6xHIS F129Y, V291 ΔScERG11::LoxP-HIS1 | This study |
| Y2649 | Y-F1/CPR | ADΔΔ PDR15::RaCPR1-6xHIS PDR5::RaCYP51-F1-6xHIS ΔScERG11::HIS1 | This study |
| Y2651 | Y-F5/CPR | ADΔΔ PDR15::RaCPR1-6xHIS PDR5::RaCYP51-F5-6xHIS ΔScERG11::HIS1 | This study |
| Y2697 | Y-F5/Y/CPR | ADΔΔ PDR15::RaCPR1-6xHIS PDR5::RaCYP51-F5-6xHIS F129Y ΔScERG11::HIS1 | This study |
| Y2695 | Y-F5/V/CPR | ADΔΔ PDR15::RaCPR1-6xHIS PDR5::RaCYP51-F5-6xHIS A291V ΔScERG11::HIS1 | This study |
| Y2693 | Y-F5/YV/CPR | ADΔΔ PDR15::RaCPR1-6xHIS PDR5::RaCYP51F5-6xHIS F129Y A291V ΔScERG11::HIS1 | This study |
| Y2653 |  | ADΔΔ proERG11::proGAL1 PDR5::RaCYP51-F1-6xHIS | This study |
| Y2655 |  | ADΔΔ proERG11::proGAL1 PDR5::RaCYP51-F5-6xHIS | This study |
| Y2659 |  | ADΔΔ proERG11::proGAL1 PDR15::RaCPR1-6xHIS PDR5::RaCYP51-F1-6xHIS | This study |
| Y2661 |  | ADΔΔ proERG11::proGAL1 PDR15::RaCPR1-6xHIS PDR5::RaCYP51-F5-6xHIS | This study |

**Table S3.** Generation times of recombinant yeast strains. Growth rates were determined to estimate strain fitness.

| **Strain** | **Generation time [h]** | |
| --- | --- | --- |
| **Y1857 (host)** | 2.20 | ±0.10 |
| **Y2300 (ScErg11)** | 2.20 | ±0.10 |
| **Y-F1** | 3.47 | ±0.15 |
| **Y-F5** | 2.10 | ±0.00 |
| **Y-F1/CPR** | 2.77 | ±0.35 |
| **Y-F5/CPR** | 2.03 | ±0.06 |
| **Y-F5/Y/CPR** | 2.07 | ±0.06 |
| **Y-F5/V/CPR** | 2.23 | ±0.06 |
| **Y-F5/YV/CPR** | 2.40 | ±0.10 |

Average of biological triplicates ± standard deviation. All strains lack the endogenous *ScERG11* gene (except the host strain Y1857).

Recombinant yeast strains Y-F5 and Y-F5/CPR, which express RaCYP51-F5 or RaCYP51-F5+CPR, respectively, grew with generation times of ~2.1 h, comparable to the host strain Y1857 and strain Y2300 which overexpresses recombinant ScErg11. Strains Y-F1 and Y-F1/CPR which express RaCYP51-F1 or RaCYP51-F1+CPR, gave generation times of 3.5 and 2.8 h, respectively. The three strains derived from Y-F5/CPR, with single F129Y or A291V mutations, or the F129Y A291V double mutation in F5, gave slightly longer generation times (≤ 18% longer) than their parental strain, with values that approached those of strain Y-F1/CPR.

**Table S4.** Detailed results of the susceptibility testing. Average of biological triplicates including ± standard deviation.

|  | **Average MIC_80_ [µM] ± standard deviation** | | | | | |
| --- | --- | --- | --- | --- | --- | --- |
| **Strain** | **Fluconazole** | **Voriconazole** | **Isavuconazole** | **Itraconazole** | **Posaconazole** | **Amphotericin B** |
| **Y1857** | 2.12± | 0.05± | 0.02± | 0.06± | 0.10± | 1.85± |
| Host | 0.20 | 0.01 | 0.01 | 0.00 | 0.03 | 0.18 |
| **Y-F1** | 2.19± | 0.14± | 0.05± | 0.02± | 0.03± | 0.65± |
| RaCYP51-F1 | 0.08 | 0.05 | 0.01 | 0.00 | 0.01 | 0.07 |
| **Y-F5** | 84.50± | 1.04± | 0.30± | 0.08± | 0.07± | 2.16± |
| RaCYP51-F5 | 9.99 | 0.02 | 0.04 | 0.02 | 0.03 | 0.37 |
| **Y-F1/CPR** | 5.22± | 0.34± | 0.10± | 0.03± | 0.05± | 1.77± |
| RaCYP51-F1+CPR | 1.16 | 0.24 | 0.05 | 0.01 | 0.01 | 0.02 |
| **Y-F5/CPR** | 446.67± | 5.19± | 0.89± | 0.19± | 0.10± | 1.82± |
| RaCYP51-F5+CPR | 39.63 | 1.32 | 0.20 | 0.01 | 0.03 | 0.06 |
| **Y-F5/Y/CPR** | 41.33± | 1.43± | 0.36± | 0.05± | 0.06± | 1.76± |
| RaCYP51-F5 F129Y+CPR | 13.99 | 0.34 | 0.12 | 0.01 | 0.02 | 0.02 |
| **Y-F5/V/CPR** | 37.50± | 0.34± | 0.30± | 0.04± | 0.04± | 1.64± |
| RaCYP51-F5 A291V+CPR | 7.50 | 0.23 | 0.14 | 0.02 | 0.02 | 0.29 |
| **Y-F5/YV/CPR** | 4.48± | 0.13± | 0.27± | 0.01± | 0.03± | 1.68± |
| RaCYP51-F5 F129Y A291V+CPR | 1.16 | 0.10 | 0.24 | 0.00 | 0.01 | 0.21 |

**Table S5**. Detailed sterol composition of RaCYP51 [%] overexpressing strains exposed to 0.1 µM voriconazole or posaconazole.

| Strain | Treatment | Lichesterol | Ergosterol | ergosta-7,22-dienol | ergosta-5,8,22,24(28)-tetraenol | 14-methyl-ergosta-7,24(28)-dienol | 2 hydroxy | 14-Methylfecosterol | ergosta-5,7-dienol | Episterol | Lanosterol | 14-methylergosta-8,24(28)-dien-3,6-diol |
| --- | --- | --- | --- | --- | --- | --- | --- | --- | --- | --- | --- | --- |
| **Y1857**  Host strain | VRC | 0.2±0.0 | 24.4±0.5 | 0.0±0.0 | 0.0±0.0 | 1.4±0.0 | 0.9±0.2 | 5.4±0.2 | 0.0±0.0 | 0.0±0.0 | 37.6±2.2 | 28.1±1.1 |
|  | POS | 0.2±0.0 | 17.7±2.2 | 0.0±0.1 | 0.0±0.0 | 1.9±0.0 | 0.9±0.3 | 5.7±0.3 | 0.0±0.0 | 0.0±0. | 36.0±1.0 | 35.4±3.3 |
|  | ctrl | 0.4±0.1 | 90.7±0.6 | 1.2±0.1 | 0.6±0.7 | 0.0±0.0 | 0.0±0.0 | 0.0±0.0 | 3.5±0.4 | 0.9±0.1 | 2.6±0.4 | 0.0±0.0 |
| **Y-F1**  RaCYP51-F1 | VRC | 0.2±0.0 | 13.6±1.0 | 1.3±0.1 | 0.0±0.0 | 2.7±0.1 | 0.0±0.0 | 5.2±0.3 | 0.0±0.0 | 0.0±0.0 | 33.4±5.8 | 43.0±6.8 |
|  | POS | 0.0±0.0 | 5.2±0.2 | 0.8±0.5 | 0.0±0.0 | 3.9±0.2 | 0.0±0.0 | 4.5±0.4 | 0.0±0.0 | 0.0±0.0 | 38.1±8.0 | 47.2±8.0 |
|  | ctrl | 0.3±0.0 | 28.9±2.5 | 0.1±0.1 | 0.0±0.0 | 1.1±0.2 | 0.2±0.2 | 4.5±0.0 | 0.1±0.1 | 0.0±0.0 | 32.9±0.5 | 30.7±2.2 |
| **Y-F5**  RaCYP51-F5 | VRC | 0.4±0.2 | 62.1±4.1 | 0.2±0.2 | 0.5±0.5 | 0.3±0.1 | 0.1±0.1 | 2.4±0.2 | 0.3±0.2 | 0.0±0.0 | 25.2±1.5 | 7.9±2.5 |
|  | POS | 0.1±0.0 | 8.1±0.6 | 0.2±0.3 | 0.0±0.0 | 3.3±0.4 | 0.5±0.0 | 5.2±0.5 | 0.0±0.0 | 0.0±0.0 | 30.8±3.0 | 49.7±3.9 |
|  | ctrl | 0.4±0.2 | 78.9±0.8 | 0.6±0.2 | 0.7±0.7 | 0.0±0.0 | 0.0±0.0 | 1.0±0.1 | 1.0±0.2 | 0.4±0.1 | 15.9±0.6 | 1.1±0.8 |
| **Y-F1/CPR**  RaCYP51-F1+CPR | VRC | 0.4±0.2 | 62.1±4.6 | 0.1±0.1 | 0.1±0.1 | 0.3±0.0 | 0.2±0.1 | 2.6±0.6 | 0.4±0.1 | 0.0±0.0 | 21.8±4.5 | 11.6±2.8 |
|  | POS | 0.1±0.0 | 7.5±1.6 | 0.4±0.6 | 0.0±0.0 | 2.6±0.3 | 0.4±0.3 | 4.2±0.3 | 0.0±0.0 | 0.0±0.0 | 25.1±2.5 | 58.6±3.8 |
|  | ctrl | 0.4±0.2 | 74.9±2.3 | 0.1±0.1 | 0.1±0.1 | 0.1±0.1 | 0.0±0.0 | 1.5±0.0 | 1.2±0.4 | 0.1±0.1 | 18.2±1.5 | 3.1±.1.2 |
| **Y-F5/CPR**  RaCYP51-F5+CPR | VRC | 0.3±0.0 | 85.6±5.5 | 0.4±0.3 | 0.0±0.0 | 0.0±0.0 | 0.2±0.1 | 0.4±0.4 | 3.0±0.7 | 0.5±0.0 | 8.7±3.5 | 0.9±1.0 |
|  | POS | 0.1±0.0 | 11.9±3.0 | 0.2±0.3 | 0.0±0.0 | 2.1±0.3 | 1.2±0.4 | 5.0±0.3 | 0.0±0.0 | 0.0±0.0 | 29.4±1.7 | 47.9±3.9 |
|  | ctrl | 0.3±0.0 | 88.6±3.9 | 1.0±0.1 | 0.1±0.1 | 0.0±0.0 | 0.0±0.0 | 0.2±0.2 | 4.9±0.6 | 0.5±0.1 | 4.4±3.4 | 0.0±0.0 |
| **Y-F5/Y/CPR**  RaCYP51-F5 F129Y+CPR | VRC | 0.3±0.0 | 84.0±1.4 | 0.4±0.0 | 0.2±0.1 | 0.0±0.0 | 0.0±0.0 | 0.6±0.1 | 3.0±0.0 | 0.40±0.0 | 10.4±1.2 | 0.8±0.3 |
|  | POS | 0.1±0.0 | 11.0±0.5 | 0.2±0.3 | 0.0±0.0 | 2.4±0.2 | 1.0±0.2 | 4.9±0.4 | 0.0±0.0 | 0.0±0.0 | 27.4±3.5 | 50.9±3.9 |
|  | ctrl | 0.3±0.1 | 88.3±0.6 | 0.6±0.1 | 0.2±0.1 | 0.0±0.0 | 0.1±0.1 | 0.0±0.0 | 5.7±1.3 | 0.7±0.1 | 4.2±0.5 | 0.0±0.0 |
| **Y-F5/V/CPR**  RaCYP51-F5 A291V+CPR | VRC | 0.3±0.0 | 67.0±7.6 | 0.0±0.0 | 0.1±0.1 | 0.3±0.1 | 0.1±0.2 | 2.2±1.3 | 0.9±0.3 | 0.0±0.0 | 18.6±3.4 | 10.1±2.8 |
|  | POS | 0.1±0.0 | 10.0±2.3 | 0.2±0.3 | 0.0±0.0 | 2.7±0.3 | 0.7±0.2 | 4.5±0.4 | 0.0±.0.0 | 0.0±0.0 | 24.8±2.0 | 55.3±4.7 |
|  | ctrl | 0.3±0.1 | 80.8±6.8 | 0.6±0.4 | 0.2±0.1 | 0.1±0.1 | 0.0±0.0 | 0.9±0.6 | 2.0±0.5 | 0.2±0.1 | 11.8±4.3 | 3.0±2.2 |
| **Y-F5/YV/CPR**  RaCYP51-F5 F129YA291V+CPR | VRC | 0.2±0.0 | 51.3±0.6 | 0.0±0.0 | 0.1±0.1 | 0.4±0.0 | 0.3±0.0 | 2.9±0.0 | 0.5±0.0 | 0.0±0.0 | 26.0±0.4 | 17.6±1.0 |
|  | POS | 0.0±0.0 | 8.6±1.8 | 0.7±0.5 | 0.0±0.0 | 2.7±0.1 | 0.4±0.1 | 4.4±0.5 | 0.0±0.0 | 0.0±0.0 | 26.0±2.1 | 55.7±4.0 |
|  | ctrl | 0.1±0.1 | 46.7±3.6 | 0.3±0.3 | 0.0±0.0 | 0.9±0.4 | 0.5±0.1 | 2.6±0.2 | 0.8±0.0 | 0. 0±0.0 | 21.1±2.3 | 26.1±4.8 |

Sterol composition of recombinant *S. cerevisiae* strains expressing RaCYP51±CPR. Pre-grown cultures were exposed to 0.1 µM of either voriconazole (VRC) or posaconazole (POS) until an OD of ~ 2 was reached. Equal amounts of DMSO were added to controls. The results present the average relative amount in % of each sterol of three independent biological replicates ± standard deviation, comprising six technical replicates in total.

**Table S6.** Similarities in protein structure of proteins homologous to RaCYP51-F1 estimated using the NCBI BLAST alignment tool.

| Organism | Annotation | Accession | % Identity | % Similarity |
| --- | --- | --- | --- | --- |
| *Rhizopus delemar* | RaCYP51-F1 | [EIE87079.1](https://www.ncbi.nlm.nih.gov/protein/EIE87079.1?report=genbank&log$=protalign&blast_rank=1&RID=VRNWPYER015) | **100** | **100** |
| *Rhizopus delemar* | RaCYP51-F5 | [EIE91884.1](https://www.ncbi.nlm.nih.gov/protein/EIE91884.1?report=genbank&log$=protalign&blast_rank=1&RID=VRN5SKPB014) | 62 | 77 |
| *Rhizopus azygosporus* | RaCYP51 ERG11_3 | [RCI01577.1](https://www.ncbi.nlm.nih.gov/protein/RCI01577.1?report=genbank&log$=prottop&blast_rank=7&RID=J8UMFCSK016) | 88 | 95 |
| *Mucor lusitanicus* | MluCYP51-F1 | [KAF1798409.1](https://www.ncbi.nlm.nih.gov/protein/KAF1798409.1?report=genbank&log$=protalign&blast_rank=12&RID=J8UMFCSK016) | 81 | 90 |
| *Aspergillus fumigatus* | AfuCyp51B | [XP_749134.1](https://www.ncbi.nlm.nih.gov/protein/XP_749134.1?report=genbank&log$=prottop&blast_rank=1&RID=J8ZSCGUN013) | 46 | 64 |
| *Aspergillus fumigatus* | AfuCyp51A | [XP_752137.1](https://www.ncbi.nlm.nih.gov/protein/XP_752137.1?report=genbank&log$=protalign&blast_rank=61&RID=JB99X5V1016) | 41 | 61 |
| *Candida albicans* | CaErg11 Sc5314 | [KHC74980.1](https://www.ncbi.nlm.nih.gov/protein/KHC74980.1?report=genbank&log$=protalign&blast_rank=3&RID=JBARD8B9016) | 41 | 60 |
| *Saccharomyces cerevisiae* | ScERG11 S288C | [NP_011871.1](https://www.ncbi.nlm.nih.gov/protein/NP_011871.1?report=genbank&log$=protalign&blast_rank=1&RID=J90AJDV4114) | 46 | 64 |
| *Homo sapiens* | HsLDM | [AAC50951.1](https://www.ncbi.nlm.nih.gov/protein/AAC50951.1?report=genbank&log$=prottop&blast_rank=3&RID=J8ZJJEM8013) | 44 | 60 |

Similarities in protein structure of proteins homologous to RaCYP51-F5 estimated using the NCBI BLAST alignment tool.

| Organism | Annotation | Accession | % Identity | % Similarity |
| --- | --- | --- | --- | --- |
| *Rhizopus delemar* | RaCYP51-F5 | [EIE91884.1](https://www.ncbi.nlm.nih.gov/protein/EIE91884.1?report=genbank&log$=protalign&blast_rank=1&RID=VRN5SKPB014) | **100** | **100** |
| *Rhizopus delemar* | RaCYP51-F1 | [EIE87079.1](https://www.ncbi.nlm.nih.gov/protein/EIE87079.1?report=genbank&log$=protalign&blast_rank=1&RID=VRNWPYER015) | 62 | 77 |
| *Rhizopus azygosporus* | RaCYP51 ERG11_2 | [RCI00353.1](https://www.ncbi.nlm.nih.gov/protein/RCI00353.1?report=genbank&log$=protalign&blast_rank=7&RID=JB8UD0PT013) | 78 | 88 |
| *Mucor lusitanicus* | MluCYP450 F5 | [KAF1801703.1](https://www.ncbi.nlm.nih.gov/protein/KAF1801703.1?report=genbank&log$=protalign&blast_rank=16&RID=JB8UD0PT013) | 75 | 84 |
| *Aspergillus fumigatus* | AfuCyp51B | [XP_749134.1](https://www.ncbi.nlm.nih.gov/protein/XP_749134.1?report=genbank&log$=protalign&blast_rank=1&RID=JB99X5V1016) | 43 | 59 |
| *Aspergillus fumigatus* | AfuCyp51A | [XP_752137.1](https://www.ncbi.nlm.nih.gov/protein/XP_752137.1?report=genbank&log$=protalign&blast_rank=61&RID=JB99X5V1016) | 39 | 56 |
| *Candida albicans* | CaERG11 Sc5314 | [KHC74980.1](https://www.ncbi.nlm.nih.gov/protein/KHC74980.1?report=genbank&log$=protalign&blast_rank=1&RID=JB9ATMHC013) | 40 | 58 |
| *Saccharomyces cerevisiae* | ScERG11 S288C | [NP_011871.1](https://www.ncbi.nlm.nih.gov/protein/NP_011871.1?report=genbank&log$=protalign&blast_rank=1&RID=JB9BW2GF013) | 43 | 61 |
| *Homo sapiens* | HsLDM | [AAC50951.1](https://www.ncbi.nlm.nih.gov/protein/AAC50951.1?report=genbank&log$=protalign&blast_rank=6&RID=JBABX67101R) | 40 | 57 |

Similarities in protein structure of proteins homologous to RaCPR1 estimated using the NCBI BLAST alignment tool.

| Organism | Annotation | Accession | % Identity | % Similarity |
| --- | --- | --- | --- | --- |
| *Rhizopus delemar* | RaCPR1 | [EIE89541.1](https://www.ncbi.nlm.nih.gov/protein/EIE89541.1?report=genbank&log$=protalign&blast_rank=11&RID=J8J17FM0016) | **100** | **100** |
| *Rhizopus delemar* | RaCPR2 | [EIE77771.1](https://www.ncbi.nlm.nih.gov/protein/EIE77771.1?report=genbank&log$=protalign&blast_rank=1&RID=J8J17FM0016) | 77 | 88 |
| *Rhizopus azygosporus* | RaCPR | [RCH88017.1](https://www.ncbi.nlm.nih.gov/protein/RCH88017.1?report=genbank&log$=protalign&blast_rank=6&RID=J8JMYMMA013) | 87 | 93 |
| *Mucor lusitanicus* | hypothetical | [KAF1800877.1](https://www.ncbi.nlm.nih.gov/protein/KAF1800877.1?report=genbank&log$=prottop&blast_rank=15&RID=J8JMYMMA013) | 80 | 90 |
| *Aspergillus fumigatus* | AfuCPRA | [KEY75941.1](https://www.ncbi.nlm.nih.gov/protein/KEY75941.1?report=genbank&log$=protalign&blast_rank=3&RID=J8MGU81C016) | 48 | 64 |
| *Candida albicans* | ScNCP1p | [XP_720425.2](https://www.ncbi.nlm.nih.gov/protein/XP_720425.2?report=genbank&log$=protalign&blast_rank=5&RID=J8MT7F0F016) | 40 | 58 |
| *Saccharomyces cerevisiae* | ScNcp1p | [PTN23602.1](https://www.ncbi.nlm.nih.gov/protein/PTN23602.1?report=genbank&log$=protalign&blast_rank=5&RID=J8N8WT1Z016) | 39 | 57 |
| *Homo sapiens* | HsCPR isoform1 | [NP_001369586.1](https://www.ncbi.nlm.nih.gov/protein/NP_001369586.1?report=genbank&log$=protalign&blast_rank=2&RID=J8NDPED1013) | 43 | 59 |

**Table S7.** Sequences of plasmids (Genes of interest highlighted in bold).

| RaCPR1 | AGGGAAATAGGCCAGGTTTTCACCGTAACACGCCACATCTTGCGAATATATGTGTAGAAACTGCCGGAAATCGTCGTGTGCACTCATGGAAAACGGTGTAACAAGGGTGAACACTATCCCATATCACCAGCTCACCGTCTTTCATTGCCATACGGAACTCCGGATGAGCATTCATCAGGCGGGCAAGAATGTGAATAAAGGCCGGATAAAACTTGTGCTTATTTTTCTTTACGGTCTTTAAAAAGGCCGTAATATCCAGCTGAACGGTCTGGTTATAGGTACATTGAGCAACTGACTGAAATGCCTCAAAATGTTCTTTACGATGCCATTGGGATATATCAACGGTGGTATATCCAGTGATTTTTTTCTCCATTTTTTTTTCCTCCTTTAGAAAAACTCATCGAGCATCAAATGAAACTGCAATTTATTCATATCAGGATTATCAATACCATATTTTTGAAAAAGCCGTTTCTGTAATGAAGGAGAAAACTCACCGAGGCAGTTCCATAGGATGGCAAGATCCTGGTATCGGTCTGCGATTCCGACTCGTCCAACATCAATACAACCTATTAATTTCCCCTCGTCAAAAATAAGGTTATCAAGTGAGAAATCACCATGAGTGACGACTGAATCCGGTGAGAATGGCAAAAGTTTATGCATTTCTTTCCAGACTTGTTCAACAGGCCAGCCATTACGCTCGTCATCAAAATCACTCGCATCAACCAAACCGTTATTCATTCGTGATTGCGCCTGAGCGAGGCGAAATACGCGATCGCTGTTAAAAGGACAATTACAAACAGGAATCGAGTGCAACCGGCGCAGGAACACTGCCAGCGCATCAACAATATTTTCACCTGAATCAGGATATTCTTCTAATACCTGGAACGCTGTTTTTCCGGGGATCGCAGTGGTGAGTAACCATGCATCATCAGGAGTACGGATAAAATGCTTGATGGTCGGAAGTGGCATAAATTCCGTCAGCCAGTTTAGTCTGACCATCTCATCTGTAACATCATTGGCAACGCTACCTTTGCCATGTTTCAGAAACAACTCTGGCGCATCGGGCTTCCCATACAAGCGATAGATTGTCGCACCTGATTGCCCGACATTATCGCGAGCCCATTTATACCCATATAAATCAGCATCCATGTTGGAATTTAATCGCGGCCTCGACGTTTCCCGTTGAATATGGCTCATTTTTTTTTCCTCCTTTACCAATGCTTAATCAGTGAGGCACCTATCTCAGCGATCTGTCTATTTCGTTCATCCATAGTTGCCTGACTCCCCGTCGTGTAGATAACTACGATACGGGAGGGCTTACCATCTGGCCCCAGCGCTGCGATGATACCGCGAGAACCACGCTCACCGGCTCCGGATTTATCAGCAATAAACCAGCCAGCCGGAAGGGCCGAGCGCAGAAGTGGTCCTGCAACTTTATCCGCCTCCATCCAGTCTATTAATTGTTGCCGGGAAGCTAGAGTAAGTAGTTCGCCAGTTAATAGTTTGCGCAACGTTGTTGCCATCGCTACAGGCATCGTGGTGTCACGCTCGTCGTTTGGTATGGCTTCATTCAGCTCCGGTTCCCAACGATCAAGGCGAGTTACATGATCCCCCATGTTGTGCACGTTGTCAGAAGTAAGTTGGCCGCAGTGTTATCACTCATGGTTATGGCAGCACTGCATAATTCTCTTACTGTCATGCCATCCGTAAGATGCTTTTCTGTGACTGGTGAGTACTCAACCAAGTCATTCTGAGAATAGTGTATGCGGCGACCGAGTTGCTCTTGCCCGGCGTCAATACGGGATAATACCGCGCCACATAGCAGAACTTTAAAAGTGCTCATCATTGGAAAACGTTCTTCGGGGCGAAAACTCTCAAGGATCTTACCGCTGTTGAGATCCAGTTCGATGTAACCCACTCGTGCACCCAACTGATCTTCAGCATCTTTTACTTTCACCAGCGTTTCTGGGTGAGCAAAAACAGGAAGGCAAAATGCCGCAAAAAAGGGAATAAGGGCGACACGGAAATGTTGAATACTCATATTCTTCCTTTTTCAATATTATTGAAGCATTTATCAGGGTTATTGTCTCATGAGCGGATACATATTTGAATGTATTTAGAAAAATAAACAAATAGGGGTCAGTGTTACAACCAATTAACCAATTCTGAACATTATCGCGAGCCCATTTATACCTGAATATGGCTCATAACACCCCTTGTTTGCCTGGCGGCAGTAGCGCGGTGGTCCCACCTGACCCCATGCCGAACTCAGAAGTGAAACGCCGTAGCGCCGATGGTAGTGTGGGGACTCCCCATGCGAGAGTAGGGAACTGCCAGGCATCAAATAAAACGAAAGGCTCAGTCGAAAGACTGGGCCTTTCGCCCGGGCTAATTGAGGGGTGTCGCCCTTCGCTGAA**CTCGTTCGAAAGACTTAATTAAAAAATGACTAGAAATAACTCACATCATCTATTAGATACAGTTGATCTAATTCTATTGGGGACTATTGGCCTAGGAACAGTTGCATGGTTCGCAAGACATCAGATTGCGAACAGACTGTTTAAGTCTGACTCAACCAATAAGTCTGAAGTCAAGGATGAAGCTAAGACACCAAAACAAGAGAGAAACTTTGTTAAGGTTATGCAACAGCAAGGTCGTAGAGTAATTTTCTTTTACGGTAGCCAAACGGGAACAGCTGAAGATTTCGCATCTAGACTTGCAAAGGAATGTACTCAGAAATACGGTGTATCTGCTATGACAGCTGATATCGAACAGTATGATCTTAGTTACCTTGATTCCGTACCTGAAGATTCATTAGTGTTCTTTATCATGGCAACCTATGGCGAGGGTGAGCCTACTGATAACGCAGTTGATTTTTGGGACTTGCTAGCGGAAGAGGTGCCTGAATTCTCGAATGATGACGGCGAAGGCAAACCATTACAAAAGCTAAGATACGTTGCTTTCGGACTAGGCAACAAGACATATGAACACTACAACGAAGTTATTAGAAAGGTTGATAACAGACTCTTGTCCTTAGGGGCAAAGCGTATCGGTGAGCGTGGAGAGGGCGATGATGATGGAACTTTAGAAGAGGACTTCCTCGCCTGGCAAGAGGAGATGTGGCCGGCCTTTTGTGAGGCTCTCGGAGTTGATGAATCAAATGCACACTCCGGACCTAGACAGGCAATTTTTAAAATTGAGGAATTGACTGCCTACGATCAAGCAAAAGTATACTTGGGTGAGATAGGTGAATGGCTTAAGGAGGGTGCTTCTATTGTTTACGACGCGAAAAGACCATACAACGCACCAATCACATCAAAGGATATTTTCAAGGCCGGTGATAGACACTGCTTGCACCTTGAGATTGACATCTCTAACACTAACTTATCATACCAAACTGGCGACCATGTCGCTATCTGGCCAACAAATAACGAAGTTGAAGTTAATAGATTGGCGAAGCTTCTAGGATTACAAAACAAGTTAGATACCGTTATTCATGTCCAATCTTTAGATCCAGCTGCGAGTAAAAAGTACCCTTTCCCAGTTCCAACCACTTACAGAGCTGTTTTCAGACATTATCTGGACATCTGTTCAGCTGTGCCAAGACAAGTCTTGATGTCATTGATCGAGTACGCTCCAACCGAAGCATCCAAAGAGGCCTTACGTAAACTAGCTACAGATAAAGATGAATATAGAGTTCACGTGGGTGATGTTACCAGAAATCTTGGTGAAGTCCTTCAAATGCTGGCCGAATCTGAAAGTTTAGAGTTGGACGGGGCCTTTTCTAGTGTCCCTTTCGATTTGGTTATCGAATCCATTTCTAGATTACAGCCTAGATACTACAGCATCAGTTCATCCTCTAAGGAAAATCCAAAAAAGATAGCTGTTACAGCTGTGACACTTCAATACACACCAGAACATGGGTCACCTAGAACAGTCTACGGTGTTAACACAAACTACTTGTGGCGTGTTCATGAAGCTGTGAACAATTTGACGCCAAATTCTGTCATCCCAGAATACAATTTGACAGGTCCAAGAGACTCCTTATTTTCACAGCAAGGAAAAGTTGCTAGAATACCTGTTCACGTTAGAAGATCTCAATTCAAGTTGCCTAGAAACCCAACTGTACCAGTGATCATGATAGGTCCAGGTACCGGTGTAGCACCATTCAGAGGTTTTGTTAGAGAACGTGCATTACAAAAAAAGGAAAACAAGCCTGTTGGTCCTACAATCTTGTTCTTCGGGTGCAGAAATAGAGCAGAGGATTTTCTGTACGAAGAGGAATGGCCAGAGTTATTCGAAGTCCTTGGTGGGGATTCTAGAATAATTACAGCTTTCTCTAGGGAAACTGAGAAAAAGGTATACGTCCAACATAGATTGATGGAAAACGGCCAAGAGATGTGGAATTTACTGGAAAAGGGTGCCTACGTTTACGTTTGCGGTGACGCAAAAAACATGGCCCGTGACGTCAACCAAACTTTCGTAAGATTCGCCCAACAATTCGGTGGTATGGATGAAAACAGATCACAAGATTATGTCAAAAACCTCCGTAACACTGGCAGATACCAGGAAGATGTGTGGAGCGGCGGCCGCCATCATCACCATCATCATTAA**CGTCAAAAGGGCGACACAAAATTTATTCTAAATGCATAATAAATACTGATAACATCTTATAGTTTGTATTATATTTTGTATTATCGTTGACATGTATAATTTTGATATCAAAAACTGATTTTCCCTTTATTATTTTCGAGATTTATTTTCTTAATTCTCTTTAACAAACTAGAAATATTGTATATACAAAAAATCATAAATAATAGATGAATAGTTTAATTATAGGTGTTCATCAATCGAAAAAGCAACGTATCTTATTTAAAGTGCGTTGCTTTTTTCTCATTTATAAGGTTAAATAATTCTCATATATCAAGCAAAGTGACAGGCGCCCTTAAATATTCTGACAAATGCTCTTTCCCTAAACTCCCCCCATAAAAAAACCCGCCGAAGCGGGTTTTTACGTTATTTGCGGATTAACGATTACTCGTTATCAGAACCGCCCAGGGGGCCCGAGCTTAAGACTGGCCGTCGTTTTACAACACAGAAAGAGTTTGTAGAAACGCAAAAAGGCCATCCGTCAGGGGCCTTCTGCTTAGTTTGATGCCTGGCAGTTCCCTACTCTCGCCTTCCGCTTCCTCGCTCACTGACTCGCTGCGCTCGGTCGTTCGGCTGCGGCGAGCGGTATCAGCTCACTCAAAGGCGGTAATACGGTTATCCACAGAATCAGGGGATAACGCAGGAAAGAACATGTGAGCAAAAGGCCAGCAAAAGGCCAGGAACCGTAAAAAGGCCGCGTTGCTGGCGTTTTTCCATAGGCTCCGCCCCCCTGACGAGCATCACAAAAATCGACGCTCAAGTCAGAGGTGGCGAAACCCGACAGGACTATAAAGATACCAGGCGTTTCCCCCTGGAAGCTCCCTCGTGCGCTCTCCTGTTCCGACCCTGCCGCTTACCGGATACCTGTCCGCCTTTCTCCCTTCGGGAAGCGTGGCGCTTTCTCATAGCTCACGCTGTAGGTATCTCAGTTCGGTGTAGGTCGTTCGCTCCAAGCTGGGCTGTGTGCACGAACCCCCCGTTCAGCCCGACCGCTGCGCCTTATCCGGTAACTATCGTCTTGAGTCCAACCCGGTAAGACACGACTTATCGCCACTGGCAGCAGCCACTGGTAACAGGATTAGCAGAGCGAGGTATGTAGGCGGTGCTACAGAGTTCTTGAAGTGGTGGGCTAACTACGGCTACACTAGAAGAACAGTATTTGGTATCTGCGCTCTGCTGAAGCCAGTTACCTTCGGAAAAAGAGTTGGTAGCTCTTGATCCGGCAAACAAACCACCGCTGGTAGCGGTGGTTTTTTTGTTTGCAAGCAGCAGATTACGCGCAGAAAAAAAGGATCTCAAGAAGATCCTTTGATCTTTTCTACGGGGTCTGACGCTCAGTGGAACGACGCGCGCGTAACTCACGTTAAGGGATTTTGGTCATGAGCTTGCGCCGTCCCGTCAAGTCAGCGTAATGCTCTGCTTAGGTGGCGGTACTTGGGTCGATATCAAAGTGCATCACTTCTTCCCGTATGCCCAACTTTGTATAGAGAGCCACTGCGGGATCGTCACCGTAATCTGCTTGCACGTAGATCACATAAGCACCAAGCGCGTTGGCCTCATGCTTGAGGAGATTGATGAGCGCGGTGGCAATGCCCTGCCTCCGGTGCTCGCCGGAGACTGCGAGATCATAGATATAGATCTCACTACGCGGCTGCTCAAACTTGGGCAGAACGTAAGCCGCGAGAGCGCCAACAACCGCTTCTTGGTCGAAGGCAGCAAGCGCGATGAATGTCTTACTACGGAGCAAGTTCCCGAGGTAATCGGAGTCCGGCTGATGTTGGGAGTAGGTGGCTACGTCACCGAACTCACGACCGAAAAGATCAAGAGCAGCCCGCATGGATTTGACTTGGTCAGGGCCGAGCCTACATGTGCGAATGATGCCCATACTTGAGCCACCTAACTTTGTTTTAGGGCGACTGCCCTGCTGCGTAACATCGTTGCTGCTCCATAACATCAAACATCGACCCACGGCGTAACGCGCTTGCTGCTTGGATGCCCGAGGCATAGACTGTACAAAAAAACAGTCATAACAAGCCATGAAAACCGCCACTGCGCCGTTACCACCGCTGCGTTCGGTCAAGGTTCTGGACCAGTTGCGTGAGCGCATTTTTTTTTCCTCCTCGGCGTTTACGCCCCGCCCTGCCACTCATCGCAGTACTGTTGTAATTCATTAAGCATTCTGCCGACATGGAAGCCATCACAGACGGCATGATGAACCTGAATCGCCAGCGGCATCAGCACCTTGTCGCCTTGCGTATAATATTTGCCCATAGTGAAAACGGGGGCGAAGAAGTTGTCCATATTGGCCACGTTTAAATCAAAACTGGTGAAACTCACCCAGGGATTGGCGCTGACGAAAAACATATTCTCAATAAACCCTTT |
| --- | --- |
| Ra  CYP51-F1 | CTGGGCGGTTCTGATAACGAGTAATCGTTAATCCGCAAATAACGTAAAAACCCGCTTCGGCGGGTTTTTTTATGGGGGGAGTTTAGGGAAAGAGCATTTGTCAGAATATTTAAGGGCGCCTGTCACTTTGCTTGATATATGAGAATTATTTAACCTTATAAATGAGAAAAAAGCAACGCACTTTAAATAAGATACGTTGCTTTTTCGATTGATGAACACCTATAATTAAACTATTCATCTATTATTTATGATTTTTTGTATATACAATATTTCTAGTTTGTTAAAGAGAATTAAGAAAATAAATCTCGAAAATAATAAAGGGAAAATCAGTTTTTGATATCAAAATTATACATGTCAACGATAATACAAAATATAATACAAACTATAAGATGTTATCAGTATTTATTATGCATTTAGAATAAATTTTGTGTCGCCCTTCGCTGAA**CTCGTTCGAAAGACTTAATTAAAAAATGGCAGTTATAAGTACATTGTTGCCTACTTTGGAGTCGATTCCACTATACGCAGTCTTAGCCTTGGGTGTTTTCGTTATCATCAATATACTAAGCCAATGGTTCGGGCCAAAAAATCCTAAGGAACCACCAGTAGTGTTCTCCTGGATCCCATTTATGGGTAATGCGATCGAATTCGGAATTAACCCTATAGCGTTCTTACAGAAGTGCCAAAAGAAATATGGTGATGTTTTCACTTTTTACATGGTTGGCAAGAGAGTTACCGTTTTCCTGAACGCCGATGGCAACCAATTCGTTTTCAACGCGAAGCAAAACCTGACATCTGCAGCGGATGCATACAACCATATGACGAAGCATGTATTCGGTCCAGAAGTGGTCTATGACGCACCTCATTCTGTCTTTATGGAACAAAAACGTTTCATTAAGGCCGGGTTGAACTCAGAGTCTTTCAGACAACACGTCCCAATGATCGTAGAGGAAGTCGAGGGATTCTTTAAAAATTACAAAAAGCCTACTGGAGCTTTCGATGCTTACCACACACTGGGAGAATTGATTATTTGTACAGCCTCCAGATGTCTTATGGGTAAGGAAATTAGAGCTTCTTTAGATGATTCAGTGGCAGGGCTCTATTACGATCTAGACCAGGGTTTCAAGCCAATCAATTTTATCTTTCCAAACTTGCCACTTCCTAGTTACAGAAAGAGAGATGTTGCAAGACAGAAAATGACAGATTTGTACTCAAGCATTATCGCTCGTAGAAAGGCTGAAAATGATTTTTCTAATGCAGACCTTTTACAAGCCTTGATGGATGCTAACTACAAGGATGGTTCAAACGTTCCTGACCACCATATTGCCGGCATGATGATTGCTGTTCTATTCGGTGGTCAACATACTTCAGCAACAACATCTGCATGGACTCTCTTAGAATTAGCAGCAAGACCAGACTTGATCAGAGACTTGAGAGAGGAACAAATTACAAAGCTTGGTTCACTGAAAGCCGATCTTACATTCGATAACCTAAAGGAATTAACATTGTTAGATTCATGCGTTAGAGAGACATTGAGACTTCATCCACCAATCTTTCAGATGATGAGAAGAGTGACCGCAAACAAAGTCGTTTTTGAAAAGACCGGACATGAAATTCCTAAGGGTAACTTCTTATGTGCTGTCCCTGGTGTTACTCAAGTGGATAGTCAGTACTTTAACGAACCTCTCAAATACGACCCACTTCGTTGGATAAACCTCACCGATCCAGTACATTCTATGGAGGCTGGGGATGACTCAAACATCGACTACGGGTTCGGTGCTGTTGGTATCTCTTCCAAAAATCCATTCTTACCATTTGGCGCCGGCAGACACAGGTGCATAGGCGAACAATTCGGTTACCTACAAATTAAGACTATAATCGCTACCATTATCAGATTATTCGATATTGAGTTGGAAGATGGAAAGGGCGTACCAAAGTCTGACTACACATCCATGGTTGTGGTACCAGAAAGACCATCTAATATCAAGTACACTTGGAGAGAGGGCGGCCGCCATCATCACCATCATCATTAA**CGTCAAAAGGGCGACACCCCCTAATTAGCCCGGGCGAAAGGCCCAGTCTTTCGACTGAGCCTTTCGTTTTATTTGATGCCTGGCAGTTCCCTACTCTCGCATGGGGAGTCCCCACACTACCATCGGCGCTACGGCGTTTCACTTCTGAGTTCGGCATGGGGTCAGGTGGGACCACCGCGCTACTGCCGCCAGGCAAACAAGGGGTGTTATGAGCCATATTCAGGTATAAATGGGCTCGCGATAATGTTCAGAATTGGTTAATTGGTTGTAACACTGACCCCTATTTGTTTATTTTTCTAAATACATTCAAATATGTATCCGCTCATGAGACAATAACCCTGATAAATGCTTCAATAATATTGAAAAAGGAAGAATATGAGCCATATTCAACGGGAAACGTCGAGGCCGCGATTAAATTCCAACATGGATGCTGATTTATATGGGTATAAATGGGCTCGCGATAATGTCGGGCAATCAGGTGCGACAATCTATCGCTTGTATGGGAAGCCCGATGCGCCAGAGTTGTTTCTGAAACATGGCAAAGGTAGCGTTGCCAATGATGTTACAGATGAGATGGTCAGACTAAACTGGCTGACGGAATTTATGCCACTTCCGACCATCAAGCATTTTATCCGTACTCCTGATGATGCATGGTTACTCACCACTGCGATCCCCGGAAAAACAGCGTTCCAGGTATTAGAAGAATATCCTGATTCAGGTGAAAATATTGTTGATGCGCTGGCAGTGTTCCTGCGCCGGTTGCACTCGATTCCTGTTTGTAATTGTCCTTTTAACAGCGATCGCGTATTTCGCCTCGCTCAGGCGCAATCACGAATGAATAACGGTTTGGTTGATGCGAGTGATTTTGATGACGAGCGTAATGGCTGGCCTGTTGAACAAGTCTGGAAAGAAATGCATAAACTTTTGCCATTCTCACCGGATTCAGTCGTCACTCATGGTGATTTCTCACTTGATAACCTTATTTTTGACGAGGGGAAATTAATAGGTTGTATTGATGTTGGACGAGTCGGAATCGCAGACCGATACCAGGATCTTGCCATCCTATGGAACTGCCTCGGTGAGTTTTCTCCTTCATTACAGAAACGGCTTTTTCAAAAATATGGTATTGATAATCCTGATATGAATAAATTGCAGTTTCATTTGATGCTCGATGAGTTTTTCTAAAAGCAGAGCATTACGCTGACTTGACGGGACGGCGCAAGCTCATGACCAAAATCCCTTAACGTGAGTTACGCGCGCGTCGTTCCACTGAGCGTCAGACCCCGTAGAAAAGATCAAAGGATCTTCTTGAGATCCTTTTTTTCTGCGCGTAATCTGCTGCTTGCAAACAAAAAAACCACCGCTACCAGCGGTGGTTTGTTTGCCGGATCAAGAGCTACCAACTCTTTTTCCGAAGGTAACTGGCTTCAGCAGAGCGCAGATACCAAATACTGTTCTTCTAGTGTAGCCGTAGTTAGCCCACCACTTCAAGAACTCTGTAGCACCGCCTACATACCTCGCTCTGCTAATCCTGTTACCAGTGGCTGCTGCCAGTGGCGATAAGTCGTGTCTTACCGGGTTGGACTCAAGACGATAGTTACCGGATAAGGCGCAGCGGTCGGGCTGAACGGGGGGTTCGTGCACACAGCCCAGCTTGGAGCGAACGACCTACACCGAACTGAGATACCTACAGCGTGAGCTATGAGAAAGCGCCACGCTTCCCGAAGGGAGAAAGGCGGACAGGTATCCGGTAAGCGGCAGGGTCGGAACAGGAGAGCGCACGAGGGAGCTTCCAGGGGGAAACGCCTGGTATCTTTATAGTCCTGTCGGGTTTCGCCACCTCTGACTTGAGCGTCGATTTTTGTGATGCTCGTCAGGGGGGCGGAGCCTATGGAAAAACGCCAGCAACGCGGCCTTTTTACGGTTCCTGGCCTTTTGCTGGCCTTTTGCTCACATGTTCTTTCCTGCGTTATCCCCTGATTCTGTGGATAACCGTATTACCGCCTTTGAGTGAGCTGATACCGCTCGCCGCAGCCGAACGACCGAGCGCAGCGAGTCAGTGAGCGAGGAAGCGGAAGGCGAGAGTAGGGAACTGCCAGGCATCAAACTAAGCAGAAGGCCCCTGACGGATGGCCTTTTTGCGTTTCTACAAACTCTTTCTGTGTTGTAAAACGACGGCCAGTCTTAAGCTCGGGCCCC |

| RaCYP51-F5 | TTAGAAAAACTCATCGAGCATCAAATGAAACTGCAATTTATTCATATCAGGATTATCAATACCATATTTTTGAAAAAGCCGTTTCTGTAATGAAGGAGAAAACTCACCGAGGCAGTTCCATAGGATGGCAAGATCCTGGTATCGGTCTGCGATTCCGACTCGTCCAACATCAATACAACCTATTAATTTCCCCTCGTCAAAAATAAGGTTATCAAGTGAGAAATCACCATGAGTGACGACTGAATCCGGTGAGAATGGCAAAAGTTTATGCATTTCTTTCCAGACTTGTTCAACAGGCCAGCCATTACGCTCGTCATCAAAATCACTCGCATCAACCAAACCGTTATTCATTCGTGATTGCGCCTGAGCGAGGCGAAATACGCGATCGCTGTTAAAAGGACAATTACAAACAGGAATCGAGTGCAACCGGCGCAGGAACACTGCCAGCGCATCAACAATATTTTCACCTGAATCAGGATATTCTTCTAATACCTGGAACGCTGTTTTTCCGGGGATCGCAGTGGTGAGTAACCATGCATCATCAGGAGTACGGATAAAATGCTTGATGGTCGGAAGTGGCATAAATTCCGTCAGCCAGTTTAGTCTGACCATCTCATCTGTAACATCATTGGCAACGCTACCTTTGCCATGTTTCAGAAACAACTCTGGCGCATCGGGCTTCCCATACAAGCGATAGATTGTCGCACCTGATTGCCCGACATTATCGCGAGCCCATTTATACCCATATAAATCAGCATCCATGTTGGAATTTAATCGCGGCCTCGACGTTTCCCGTTGAATATGGCTCATATTCTTCCTTTTTCAATATTATTGAAGCATTTATCAGGGTTATTGTCTCATGAGCGGATACATATTTGAATGTATTTAGAAAAATAAACAAATAGGGGTCAGTGTTACAACCAATTAACCAATTCTGAACATTATCGCGAGCCCATTTATACCTGAATATGGCTCATAACACCCCTTGTTTGCCTGGCGGCAGTAGCGCGGTGGTCCCACCTGACCCCATGCCGAACTCAGAAGTGAAACGCCGTAGCGCCGATGGTAGTGTGGGGACTCCCCATGCGAGAGTAGGGAACTGCCAGGCATCAAATAAAACGAAAGGCTCAGTCGAAAGACTGGGCCTTTCGCCCGGGCTAATTAGGGGGTGTCGCCCTTCGCTGAA**CTCGTTCGAAAGACTTAATTAAAAAATGGCTATCATTTCCACATTATTGTCAGAGACATCACTATCTAGCCTGTTGTACAAGGCCATCGTTTTCTTGGCAACTTACTACGTTATACACTTACTATCTCAGTTCATCCAACCAAAAGATCCTAAAGCCGTCCCATTAGTGCCATCTTGGATTCCTTTCTTTGGTAATGCAATCGAGTTTGGTAAAAACCCAATCGAATTCTTACAAACATGTCAAAAGAAATACGGTGATGTTTTCACTTTCAGACTGCTTAACAAGAGAGTTACAGCGTGTCTTGGTCCTGATGGTAATCAATTCGTTTTCAACGCGAAACAAGAGGTTGCATCAGCCGCAGCAGCATATAACCACATGACTAAGTACGTATTCGGCAACGATATAGTTTTTGATACAGCTCATTCTGTGTTCATGGAACAAAAGAGAATGATAAAGTCAGGATTGAACATTGAAGCCTTTAGAAAAGATGTCCCATTGATAATTGAGGAATGTGACGCATTTTTCGACGGTCTAGAGCCTCAGGGTGAAATGGACTTGTACAAGATGTTCGGAACACTAATCATTTACACAGCATCTAGAACTTTACTAGGACCAGAAATCAGACAAGCTTTAGATTCAGGTGTGTCTGAGTTGTACTACGATCTAGATCAAGGGTTCAGACCAATTAACTTCATGTTTCCTAACTTGCCACTTCCTTCTTACAGACGTAGAGATGAAGCTAGAGAAAAGTTAGCAAAGATTTACGCTGGTATTATCCAAAAGCGTAGGCAGAGTGAACATGGTGAATCAGATTTGTTGCAAACTCTCATTGAGGCAAGATACAAGGATGGATCAGCCGTTCCAGATGCTCAAATCTGCGGAATTTTGACTGCCGCTTTATTTGGTGGGCAACACACATCTAGCACAACGGCTGCTTGGACAATACTCGAATTAGCACAAAGACCAGACATCATTCAAGCTTTGAGACAAGAGATGATTACCCAGTGCGGTTCACTTGAAGTTGACTTTACTTACGATCATCTCGAAAGATTGACCTTGCTGGAACATGTCGTTAAGGAAACTTTAAGACTTCATCCACCAATTTTCAATATGATGCGTAGAGTCGTAGCACCTAAGTGCGTATTCTCGGGCAGAGAAATCCCTCAGGGCGACTATTTGCTCGCCGCTCCAGGGGTGACACAATTGGACCCTCATTACTTTCACCAACCAACAGTGTGGGACCCATACAGATGGTCACAATTGAAAGATCCAGTTCATCAGCTTGAGCAAGGTGAAGATGCAAACGCTGATTACGGCTTTGGCGTTGTTGGTATATCCTCCAAGTCTCCATTCCTTCCATTTGGTGCGGGCAGACATAGATGTATTGGGGAAAAGTTCGGTTACCTTCAGCTAAAAACTATCATCGGTACCTTCGTAAAGAGATTCGATGTAGAGCCATTAAGTCACACCGTGCCTAAGCCAGATTATACATCTATGGTCGTTGTTCCTGAAAATTCTCATATCCGTTATAGAGCTAGAAAACAGGGCGGCCGCCATCATCACCATCATCATTAA**CGTCAAAAGGGCGACACAAAATTTATTCTAAATGCATAATAAATACTGATAACATCTTATAGTTTGTATTATATTTTGTATTATCGTTGACATGTATAATTTTGATATCAAAAACTGATTTTCCCTTTATTATTTTCGAGATTTATTTTCTTAATTCTCTTTAACAAACTAGAAATATTGTATATACAAAAAATCATAAATAATAGATGAATAGTTTAATTATAGGTGTTCATCAATCGAAAAAGCAACGTATCTTATTTAAAGTGCGTTGCTTTTTTCTCATTTATAAGGTTAAATAATTCTCATATATCAAGCAAAGTGACAGGCGCCCTTAAATATTCTGACAAATGCTCTTTCCCTAAACTCCCCCCATAAAAAAACCCGCCGAAGCGGGTTTTTACGTTATTTGCGGATTAACGATTACTCGTTATCAGAACCGCCCAGGGGGCCCGAGCTTAAGACTGGCCGTCGTTTTACAACACAGAAAGAGTTTGTAGAAACGCAAAAAGGCCATCCGTCAGGGGCCTTCTGCTTAGTTTGATGCCTGGCAGTTCCCTACTCTCGCCTTCCGCTTCCTCGCTCACTGACTCGCTGCGCTCGGTCGTTCGGCTGCGGCGAGCGGTATCAGCTCACTCAAAGGCGGTAATACGGTTATCCACAGAATCAGGGGATAACGCAGGAAAGAACATGTGAGCAAAAGGCCAGCAAAAGGCCAGGAACCGTAAAAAGGCCGCGTTGCTGGCGTTTTTCCATAGGCTCCGCCCCCCTGACGAGCATCACAAAAATCGACGCTCAAGTCAGAGGTGGCGAAACCCGACAGGACTATAAAGATACCAGGCGTTTCCCCCTGGAAGCTCCCTCGTGCGCTCTCCTGTTCCGACCCTGCCGCTTACCGGATACCTGTCCGCCTTTCTCCCTTCGGGAAGCGTGGCGCTTTCTCATAGCTCACGCTGTAGGTATCTCAGTTCGGTGTAGGTCGTTCGCTCCAAGCTGGGCTGTGTGCACGAACCCCCCGTTCAGCCCGACCGCTGCGCCTTATCCGGTAACTATCGTCTTGAGTCCAACCCGGTAAGACACGACTTATCGCCACTGGCAGCAGCCACTGGTAACAGGATTAGCAGAGCGAGGTATGTAGGCGGTGCTACAGAGTTCTTGAAGTGGTGGGCTAACTACGGCTACACTAGAAGAACAGTATTTGGTATCTGCGCTCTGCTGAAGCCAGTTACCTTCGGAAAAAGAGTTGGTAGCTCTTGATCCGGCAAACAAACCACCGCTGGTAGCGGTGGTTTTTTTGTTTGCAAGCAGCAGATTACGCGCAGAAAAAAAGGATCTCAAGAAGATCCTTTGATCTTTTCTACGGGGTCTGACGCTCAGTGGAACGACGCGCGCGTAACTCACGTTAAGGGATTTTGGTCATGAGCTTGCGCCGTCCCGTCAAGTCAGCGTAATGCTCTGCTT |
| --- | --- |

**Table S8.** Oligonucleotides used in the present study to create and analyse recombinant yeast strains.

| Name | Sequence | Purpose |
| --- | --- | --- |
| PDR5Fv2 | GCAGTCCCTTACATAGTACACAAC | Creation of PDR5::GOI cassette, *Fragment 1 (upstream)* |
| pABC3-PacI_R | CATTTTTTAATTAAGTCTTTCGAACGAGCGG | Creation of PDR5::GOI cassette, *Fragment 1 (upstream)* |
| pABC3-PacI_F | CCGCTCGTTCGAAAGACTTAATTAAAAAATG | Creation of PDR5::GOI cassette, *Fragment 2 (ORF)* |
| Not1-6xHis-R | GAATTTAATGATGATGGTGATGATGGCGGCCGCC | Creation of PDR5::GOI cassette, *Fragment 2 (ORF)* |
| Forward Not1-6xHis | GGCGGCCGCCATCATCACCATCATCATTAAATTC | Creation of PDR5::GOI cassette, *Fragment 3 (downstream)* |
| PDR5 288DS_R | CCGTAAGGCACAGTTAAGAAATAATG | Creation of PDR5::GOI cassette, *Fragment 3 (downstream)* |
| PDR5Fv3 | TCGCATTCTGCGCCTTCGAGCAC | *Amplification of PDR5::GOI transformation cassette (Fusion PCR of fragments 1, 2, 3)* |
| PDR5_186DS_R | TTCGGACATTGAACTTTGATTTATC | *Amplification of PDR5::GOI transformation cassette (Fusion PCR of fragments 1, 2, 3)* |
| PDR5Fv2 | GCAGTCCCTTACATAGTACACAAC | *Amplification of extended cassette (size and sequence control):* |
| PDR5 288DS_R | CCGTAAGGCACAGTTAAGAAATAATG | *Amplification of extended cassette (size and sequence control):* |
| PDR5US126 | TGTGTTAGTTATCACTCGACTTTG | *Additional sequencing primers for RDR5 locus* |
| PGK1_Rev | TCGGATAAGAAAGCAACACCTGG | *Additional sequencing primers for RDR5 locus* |
| ScHIS1_1894R | TTATACACGACAATTAGAAATTTC | *Additional sequencing primers for RDR5 locus* |
| PDR5_412DS_R | AAAGATCCGATTATACTTACCCAC | *Additional sequencing primers for RDR5 locus* |
| PDR15-PDR5US_F | TTTGTTCGCGCAAAGAGCAAGAACATTGGTTGCTTTACTTATTATTTTCAGGGTCGCTTCGCATTCTGCGCCTTCGAGCAC | *Creation of PDR15::GOI cassette, Fragment 1* |
| pABC3-PacI_R | CATTTTTTAATTAAGTCTTTCGAACGAGCGG | *Creation of PDR15::GOI cassette, Fragment 1 upstream* |
| pABC3-PacI_F | CCGCTCGTTCGAAAGACTTAATTAAAAAATG | *Creation of PDR15::GOI cassette, Fragment 2 ORF* |
| Not1-6xHis-R | GAATTTAATGATGATGGTGATGATGGCGGCCGCC | *Creation of PDR15::GOI cassette, Fragment 2 ORF* |
| Forward Not1-6xHis | GGCGGCCGCCATCATCACCATCATCATTAAATTC | *Creation of PDR15::GOI cassette, Fragment 3 downstream* |
| PDR15-Ura3_R | CAGTAGAGAGAATAGAATATAATAAAAAGATAATATAACTAAAAAAAAGGAAAATAACGTCATTAGTTTTGCTGGCCGCATCTTCTC | *Creation of PDR15::GOI cassette, Fragment 3 downstream* |
| PDR15USF_m | GTCACGCCGCCGAACTGCAGCGCGC | *Amplification of PDR15::GOI transformation cassette (Fusion PCR of fragments 1, 2, 3)* |
| PDR15DSR_m | GATGGAATAATCCAGTTCGACTCTG | *Amplification of PDR15::GOI transformation cassette (Fusion PCR of fragments 1, 2, 3)* |
| MRP20_697ORF_f | CGAGAGATAGACAATAAGCGAGAG | *Amplification of extended cassette (size and sequence control from gDNA of selected strains)* |
| TRS120_3781ORF frc | TACGAGTTTAGCGTTTGCATCTCC | *Amplification of extended cassette (size and sequence control from gDNA of selected strains)* |
| pABC3-PacI_R | CATTTTTTAATTAAGTCTTTCGAACGAGCGG | *PDR5/PDR15 integration control colony PCR* |
| pABC-PAC1R | CATTTTTTAATTAAGTCTTTCGAACGAGCGG | *Additional sequencing primers for RDR15 locus* |
| PDR5US126 | TGTGTTAGTTATCACTCGACTTTG | *Additional sequencing primers for RDR15 locus* |
| RoCPR1at_741F | ATGCACACTCCGGACCTAGACAGG | *Additional sequencing primers for RDR15 locus* |
| PGK1_Rev | TCGGATAAGAAAGCAACACCTGG | *Additional sequencing primers for RDR15 locus* |
| ScErg11_US-773_F | GCAACAATGGGCGGTTGTTTAGAG | *Primers for deletion of endogenous CYP51 Transformation cassette* |
| ScErg11DS346R_Δ | GACTGCTTTATTTCTGCTTGGCCTG | *Primers for deletion of endogenous CYP51 Transformation cassette* |
| ScErg11_US-801 | GCCGCCTGTCCCGTACAGACGAAC | *Amplification of extended cassette (size and sequence control from gDNA of selected strains)* |
| ScERG11-ter-rev | CACTATAAAAAGCAGTAATGAATCT | *Amplification of extended cassette (size and sequence control from gDNA of selected strains)* |
| ScHIS1_ORF27_R | ATCGGTTAGATGGTTCACCAAATCC | *Additional sequencing primers for ERG11 locus* |
| ScHIS1_1894R | TTATACACGACAATTAGAAATTTC | *Additional sequencing primers for ERG11 locus* |
| pfHIS1-3 | TTGCCGATTTGGAAGGTACT |  |
| RaCYP51_F5 Y129-F | CGTATTCGGCAACGATATAGTTTATGATACAGCTCATTCTGTGTTCAT | *Mutagenic primers for F129Y and A291V substitutions in RaCYP51-F5* |
| RaCYP51_F5 Y129-R | ATGAACACAGAATGAGCTGTATCATAAACTATATCGTTGCCGAATACG | *Mutagenic primers for F129Y and A291V substitutions in RaCYP51-F5* |
| RaCYP51_F5 V291-F | CAAATCTGCGGAATTTTGACTGCCGTTTTATTTGGTGGGCAACACACATCT | *Mutagenic primers for F129Y and A291V substitutions in RaCYP51-F5* |
| RaCYP51_F5 V291-R | AGATGTGTGTTGCCCACCAAATAAAACGGCAGTCAAAATTCCGCAGATTTG | *Mutagenic primers for F129Y and A291V substitutions in RaCYP51-F5* |
| Up_F_F1 | ATTAACTCGTACATTTCTTACTCG |  |
| Dw_R_F1 | TACCTTTGAGATAATGGTGGCTT |  |
| Up_F_F5 | ATCGCTCACCATTACGCCTT |  |
| Dw_R_F5 | TGACAAGCTCAAGCCTGGGAT |  |
| pyrF_RC | TAGTCATGCGTCCAGTTTCTGT |  |
| Templ_F_F1 | CACCTTTTATATGGTTGGCCGACGTGTTACCGTCTTCTTCCTCCATAAGAATTTGACAG |  |
| Templ_R_F1 | CTTTGCGTTAAAGACAAATTGATTACCCTCAGCGCCCATGATAAAACGAAGATGTGGCTGTC |  |
| Templ_F_F5 | TCGTCTCTTAAACAAGCGCGTCACTGCATGCCTTAGCCTCCTCCATAAGAATTTGACAG |  |
| Templ_R_F5 | GTTTTGTTTTGCATTAAATACGAATTGGTTACCATCAGTGATAAAACGAAGATGTGGCTGTC |  |
| F1_gRNA1 | CGACGTGTTACCGTCTTCTTGGG |  |
| F5_gRNA1 | ATTGGTTACCATCAGGGCTAAGG |  |

**Table S9.** Primers and crRNAs used to modify *R. microsporus*. Red sequences indicate the micro-homology tails added to the *pyrF* gene for the recombination with *CYP51-F1* and *CYP51-F5*.

| **Primer name** | **Sequence 5’→ 3’** |
| --- | --- |
| Up_F_F1 | ATTAACTCGTACATTTCTTACTCG |
| Dw_R_F1 | TACCTTTGAGATAATGGTGGCTT |
| Up_F_F5 | ATCGCTCACCATTACGCCTT |
| Dw_R_F5 | TGACAAGCTCAAGCCTGGGAT |
| pyrF_RC | TAGTCATGCGTCCAGTTTCTGT |
| Templ_F_F1 | CACCTTTTATATGGTTGGCCGACGTGTTACCGTCTTCTTCCTCCATAAGAATTTGACAG |
| Templ_R_F1 | CTTTGCGTTAAAGACAAATTGATTACCCTCAGCGCCCATGATAAAACGAAGATGTGGCTGTC |
| Templ_F_F5 | TCGTCTCTTAAACAAGCGCGTCACTGCATGCCTTAGCCTCCTCCATAAGAATTTGACAG |
| Templ_R_F5 | GTTTTGTTTTGCATTAAATACGAATTGGTTACCATCAGTGATAAAACGAAGATGTGGCTGTC |
| **crRNA name** | Sequence 5’ → 3 |
| F1_gRNA1 | CGACGTGTTACCGTCTTCTTGGG |
| F5_gRNA1 | ATTGGTTACCATCAGGGCTAAGG |

**Table S10**. *Rhizopus microsporus* strains used in this study.

| **Species** | **Parental Strain** | **Name** | **Genotype** | **Markers** |
| --- | --- | --- | --- | --- |
| *R. microsporus* | UM1 (pyrF-) ATCC11559 | RM1 | ΔCYP51-F5 | pyrF+ leuA+ |
| *R. microsporus* | UM1 (pyrF-) ATCC11559 | RM2 | ΔCYP51-F5 | pyrF+ leuA+ |
| *R. microsporus* | UM1 (pyrF-) ATCC11559 | RM3 | ΔCYP51-F1 | pyrF+ leuA+ |
| *R. microsporus* | UM1 (pyrF-) ATCC11559 | RM4 | ΔCYP51-F1 | pyrF+ leuA+ |

## Supplementary Material Figures


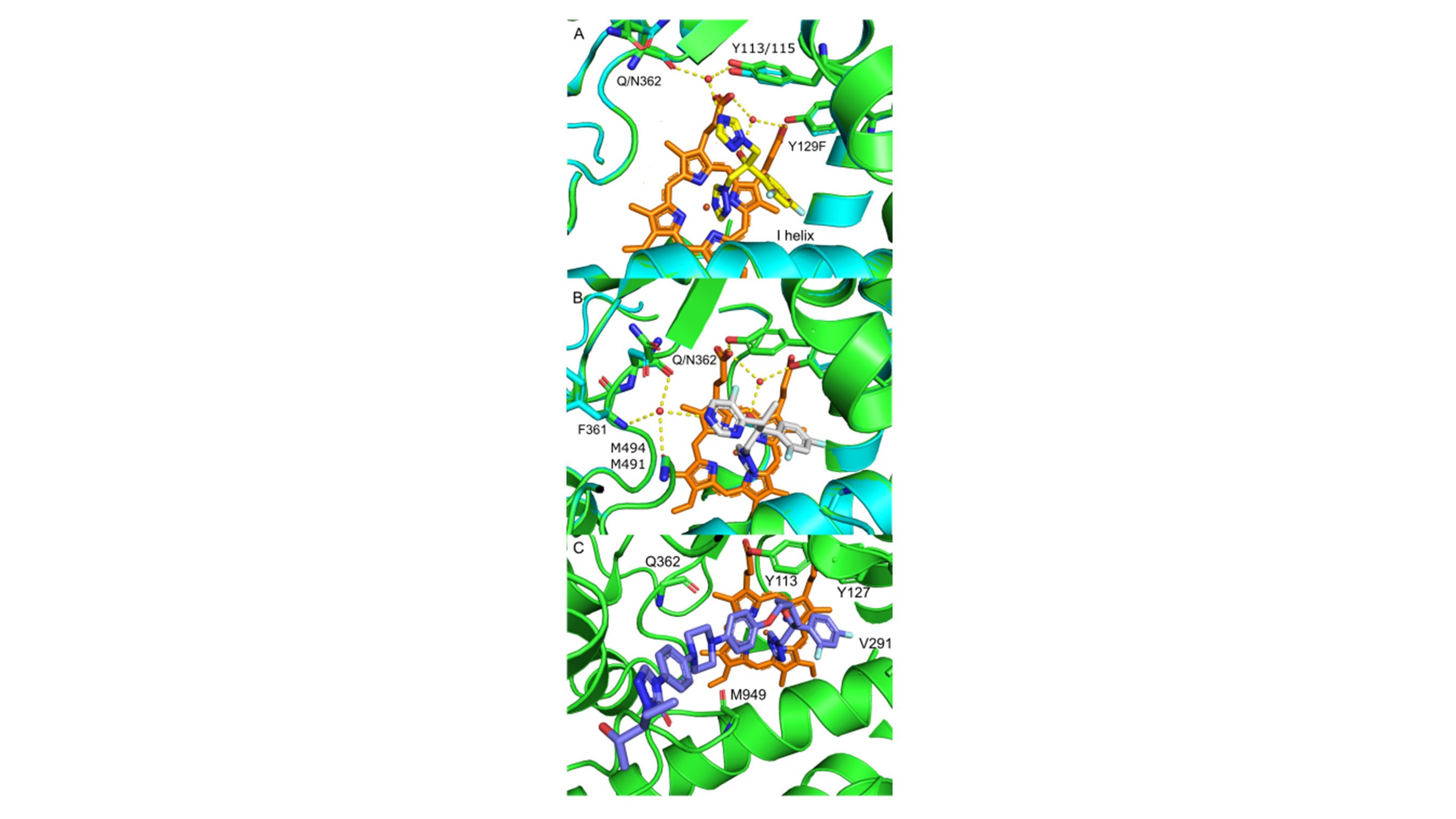


**Fig S1.** Models of RaCYP51-F1 and RaCYP51-F5 complexed with fluconazole, voriconazole and posaconazole. **(A)** Putative water-mediated hydrogen bond network between fluconazole (yellow) and RaCYP51-F1 (green) Q362 and RaCYP51-F5 (cyan) N362 and Y113 and Y115 respectively. Water molecules derived from the ScCYP51 crystal structure (PDB ID: 4WMZ) (2). **(B)** Putative water-mediated hydrogen bond network between voriconazole (white) and RaCYP51-F1 (green) F361 and RaCYP51-F5 (cyan) F361, Q362 and N362, and M494 and M491 respectively. Water molecules derived from the ScCYP51 crystal structure (PDB ID: 5HS1) (4). **(C)** Posaconazole (purple) bound to RaCYP51-F1 spanning both the active site and substrate channel. Heme shown as orange sticks, waters as red spheres, hydrogen bounds as yellow dashed lines; oxygens shown in red, nitrogen in blue, fluorine as light blue. Parts of the enzyme have been hidden for better visualization.


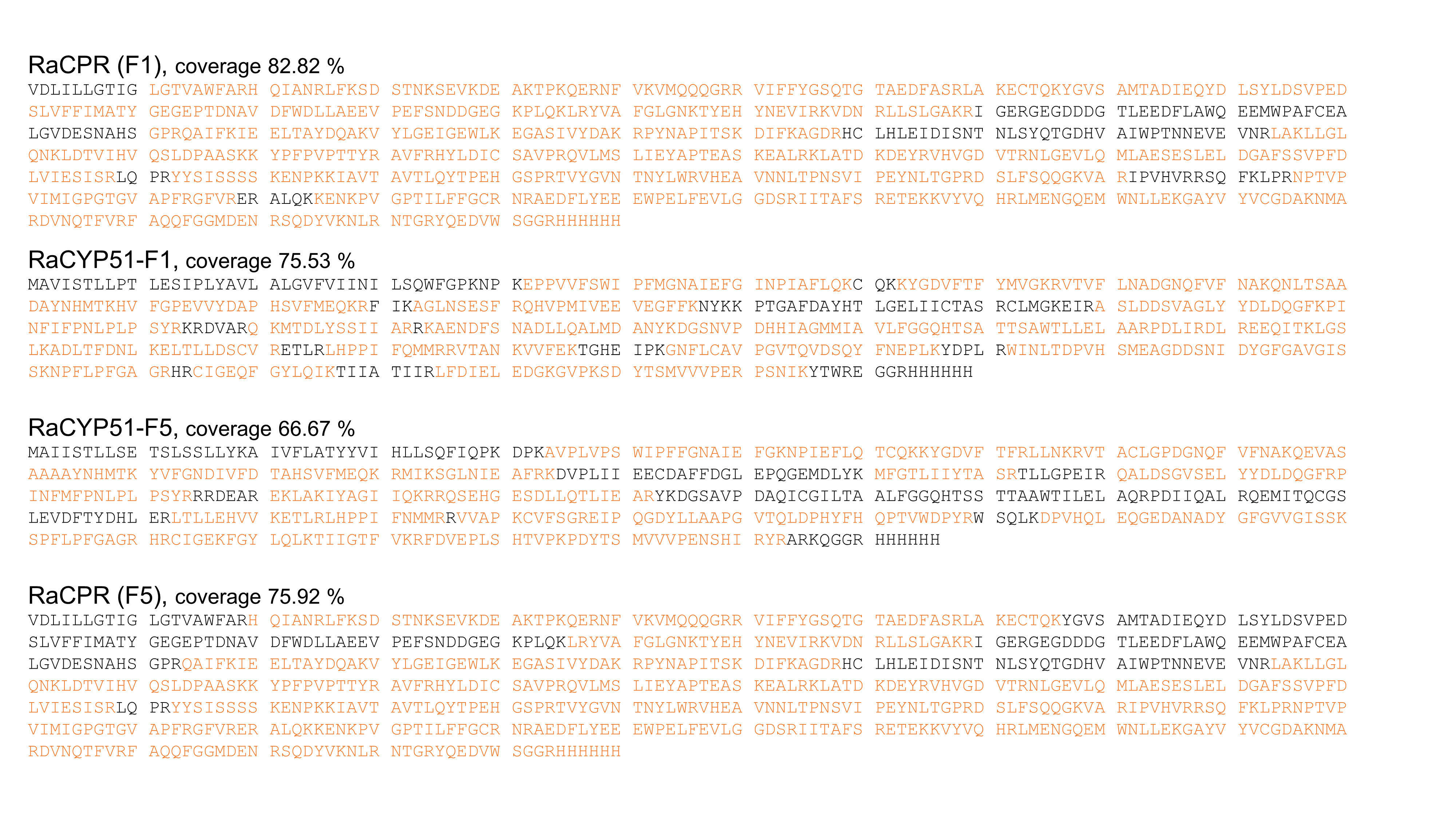


**Fig S2.** Sequence coverage highlighted in orange of SDS-PAGE separated protein bands detected by mass spectrometry of tryptic fragments.


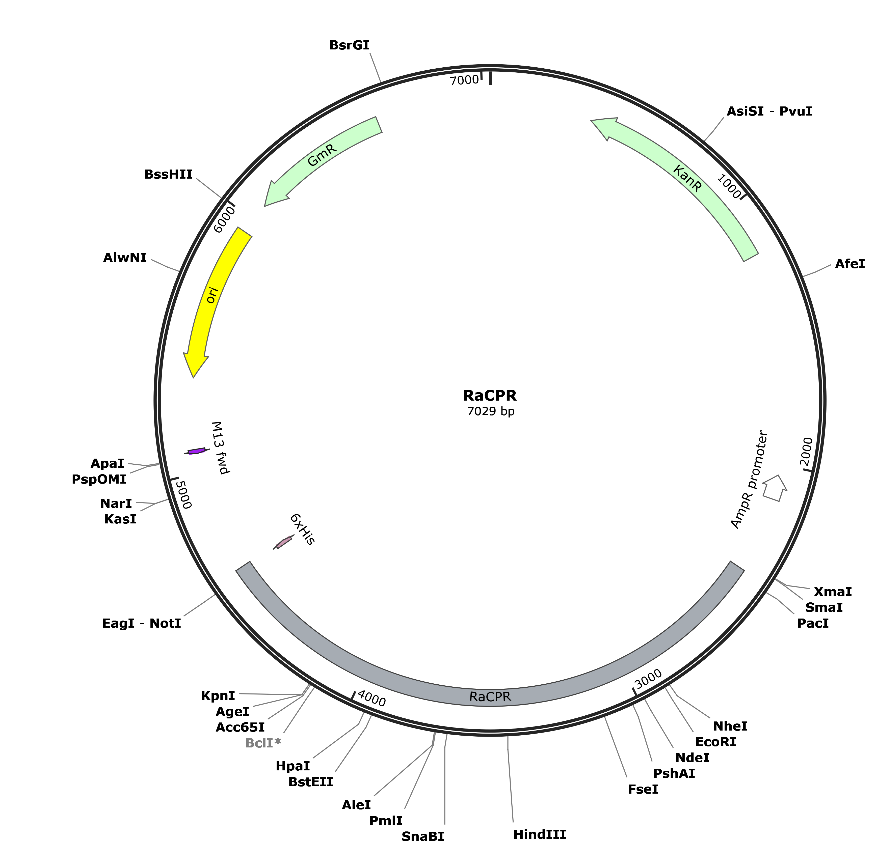


**Fig S3.** Map of plasmid bearing synthetic codon optimized, HIS-tagged *R. arrhizus*. Cognate reductase gene developed and supplied by ATUM service. Detailed sequence is given in **S7 Table**.


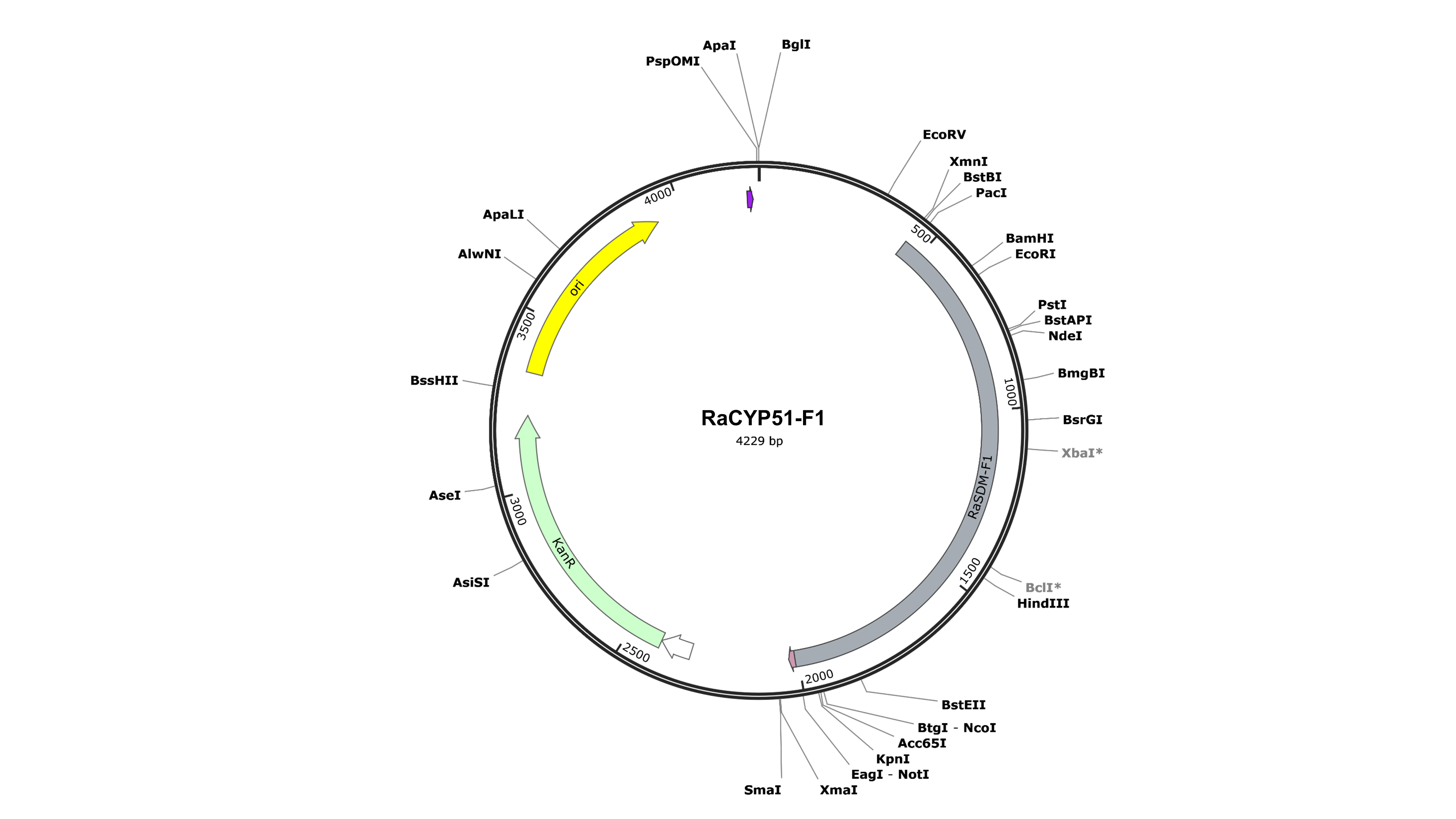


**Fig S4.** Map of plasmid bearing synthetic codon optimized, HIS-tagged *R. arrhizus* CYP51-F1. Gene developed and supplied by ATUM service. Detailed sequence is given in **S7 Table**.


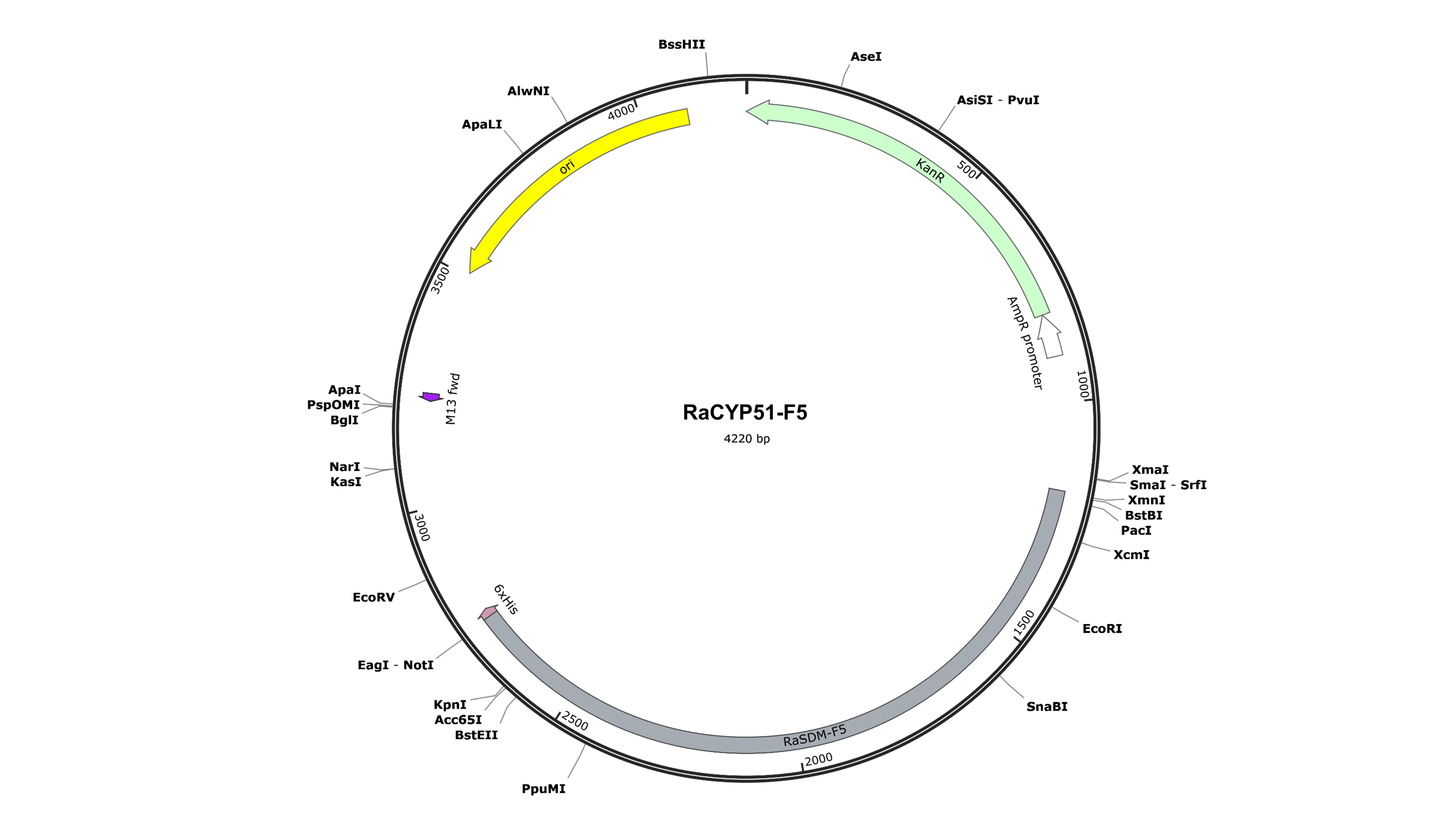


**Fig S5.** Map of plasmid bearing synthetic optimized, HIS-tagged *R. arrhizus* CYP51-F5. Gene developed and supplied by ATUM. Detailed sequence is given in **S7 Table**.

**
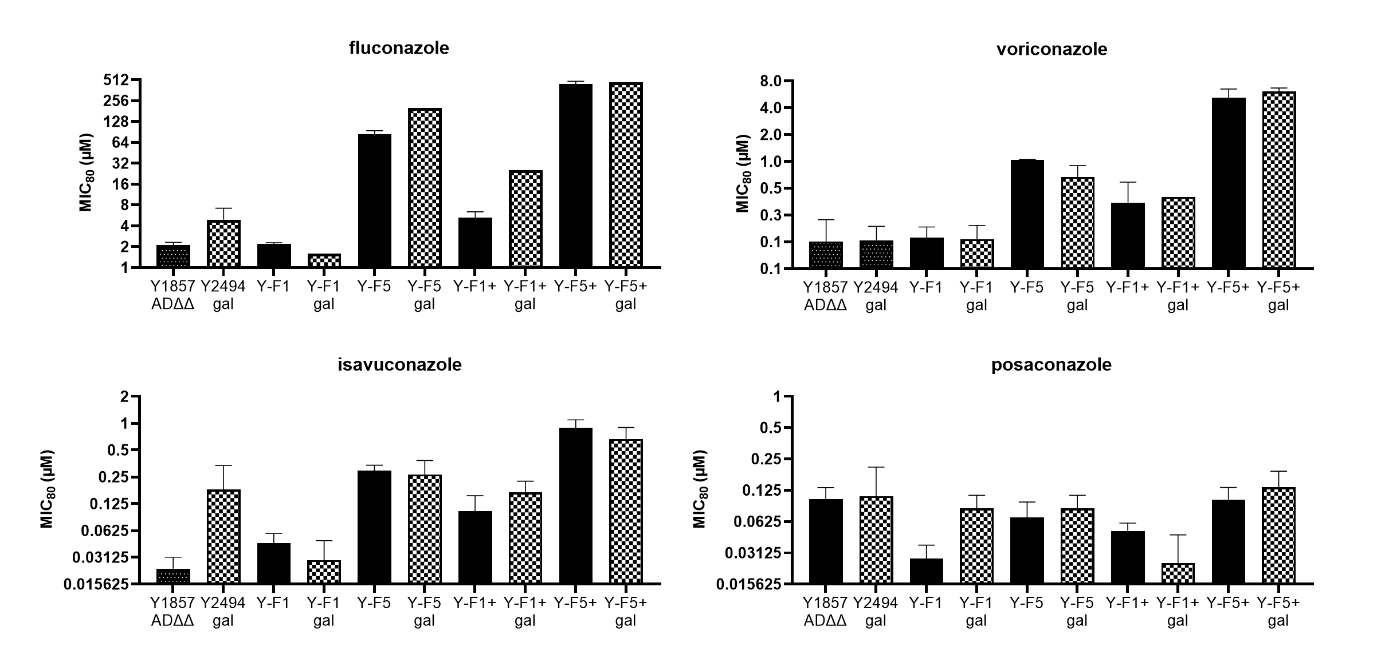
**

**Fig S6.** Susceptibility comparison of RaCYP51-F1 and RaCYP-F5±CPR overexpressed in ADΔΔ or ADΔΔgal host strain. Every two pairs of bars represent RaCYP51 overexpressed in ADΔΔ (solid fill) vs. ADΔΔgal (dotted fill). There were only minor variances between the MICs (within the two-fold variation tolerated). For strain information see **S2 Table**. Bars show results from three biological replicates including standard deviation. Abbreviation: + CPR (+), gal promoter (gal).


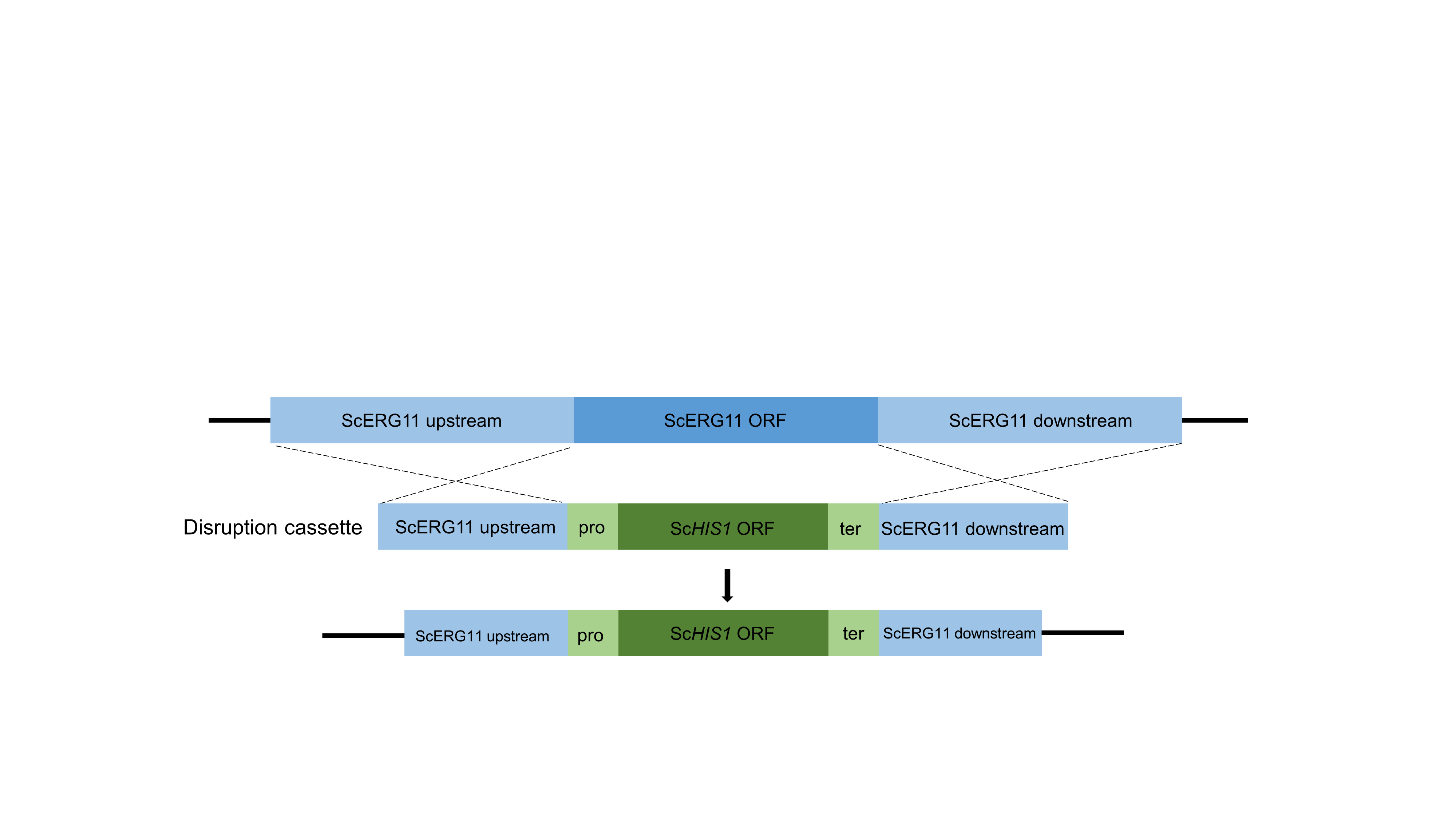


**Fig S7.** Scheme for the deletion of endogenous *S. cerevisiae* *ERG11* (following Monk et al. (3)).

**
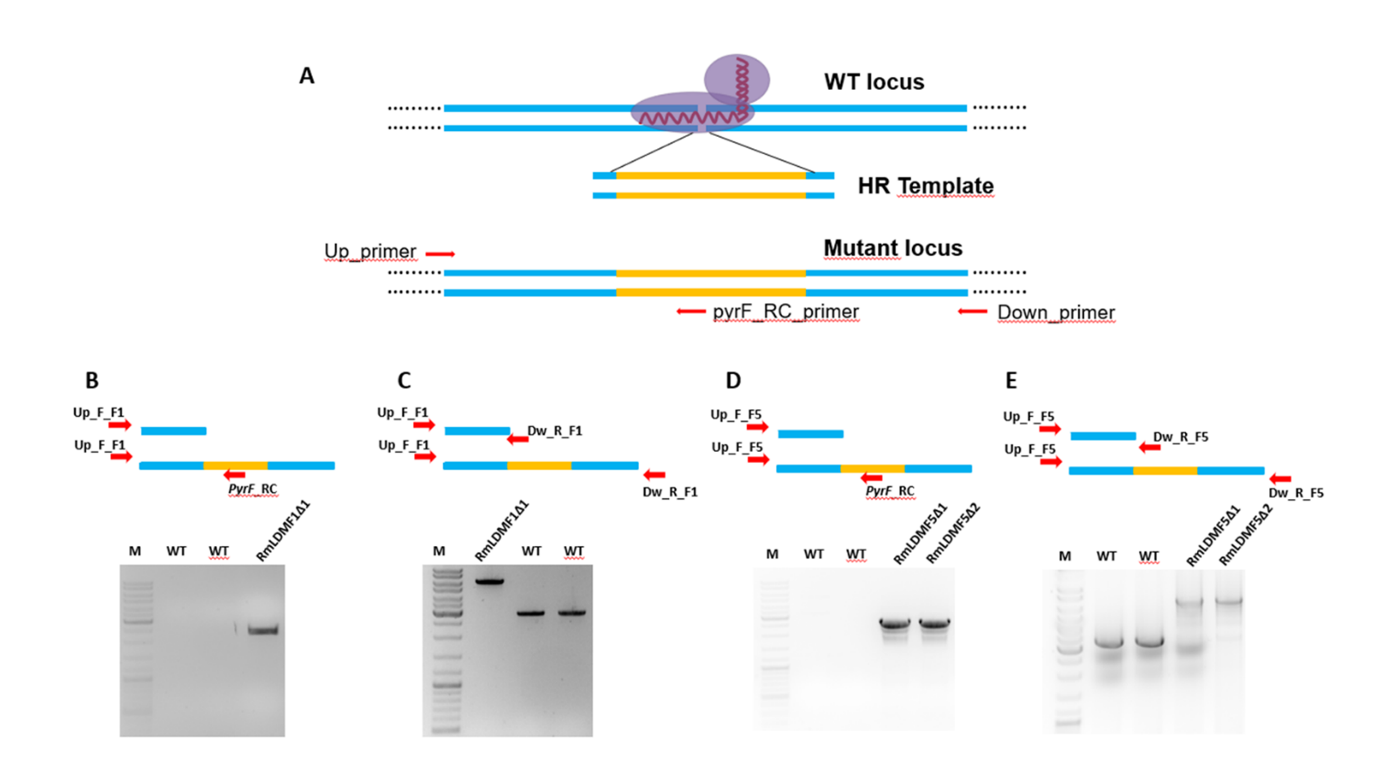
**

**Fig S8.** Generation of RmCYP51-F1 (=RmLDM-F1) and RmCYP51-F5 (=RmLDM-F5) mutant strains in *R. microsporus*. **(A)** Schematic representation of the strategy used for the targeted gene disruption. Primers used to check the disruption and the homokaryosis are indicated as red arrows. **(B)** Targeted disruption validation by PCR using the Up_F_F1 primer and pyrF RC primer for the *RmCYP51-F1* gene. A band of the expected size of 2.3 kb confirmed the integration. **(C)** PCR amplification of the *RmCYP51-F1* *locus* using the Up_F_F1 primer and the Down_R_F1 primer. Expected fragment size from wild type and disrupted *RmCYP51-F1* loci were 2.8 kb and 6.3 kb in length, respectively. **(D)** Targeted disruption validation by PCR using the Up_F_F5 primer and pyrF_RC primer for the *RmCYP51-F5* gene. A band of 2.7 kb confirmed the integration **(E)** PCR amplification of the *RmCYP51-F5* locus using the Up_F_F5 primer and the Down_R_F5 primer. Expected fragment size from wild-type and disrupted *RmCYP51-F5* locus were 3.2 kb and 6.7 kb in length, respectively.

**References**

1. Lamping E, Monk BC, Niimi K, Holmes AR, Tsao S, Tanabe K, et al. Characterization of three classes of membrane proteins involved in fungal azole resistance by functional hyperexpression in *Saccharomyces cerevisiae*. Eukaryot Cell. 2007;6(7):1150-65.

2. Sagatova AA, Keniya MV, Wilson RK, Monk BC, Tyndall JD. Structural insights into binding of the antifungal drug fluconazole to *Saccharomyces cerevisiae* lanosterol 14alpha-demethylase. Antimicrob Agents Chemother. 2015;59(8):4982-9.

3. Monk BC, Keniya MV, Sabherwal M, Wilson RK, Graham DO, Hassan HF, et al. Azole Resistance Reduces Susceptibility to the Tetrazole Antifungal VT-1161. Antimicrob Agents Chemother. 2019;63(1):e02114-18.

4. Sagatova AA, Keniya MV, Wilson RK, Sabherwal M, Tyndall JD, Monk BC. Triazole resistance mediated by mutations of a conserved active site tyrosine in fungal lanosterol 14α-demethylase. Scientific reports. 2016;6:26213.
